# Estrogen-induced miR-196a elevation promotes tumor growth and metastasis via targeting SPRED1 in breast cancer

**DOI:** 10.1186/s12943-018-0830-0

**Published:** 2018-04-23

**Authors:** Cheng-Fei Jiang, Zhu-Mei Shi, Dong-Mei Li, Ying-Chen Qian, Yi Ren, Xiao-Ming Bai, Yun-Xia Xie, Lin Wang, Xin Ge, Wei-Tao Liu, Lin-Lin Zhen, Ling-Zhi Liu, Bing-Hua Jiang

**Affiliations:** 1 The First Affiliated Hospital of Zhengzhou University, Zhengzhou University, Zhengzhou, 450052 Henan China; 2 Key Laboratory of Human Functional Genomics of Jiangsu Province, State Key Lab of Reproductive Medicine, Jiangsu Key Laboratory of Cancer Biomarkers, Prevention, and Treatment Department of Pathology, Cancer Center, Department of Pathology, Nanjing Medical University, 140 Hanzhong Road, Nanjing, China; 3 Department of Neurosurgery, The First Affiliated Hospital of Nanjing Medical University, 300 Guangzhou Road, Nanjing, China; 4 Department of Pharmacology, Guangxi Institute of Chinese Medicine and Pharmaceutical Science, Nanning, 530022 People’s Republic of China; 5 Department of Breast and Thyroid Surgery, Huai’an First People’s Hospital, Nanjing Medical University, 6 Beijing Road West, Huai’an, China; 6 Department of Pathology, University of Iowa, 25 S. Grand Avenue, Iowa City, USA

**Keywords:** Estrogen, miR-196a, SPRED1, Breast cancer, Estrogen receptors

## Abstract

**Background:**

Estrogen plays a critical role in breast cancer (BC) progression through estrogen receptor (ER)-mediated gene regulation. Emerging studies suggest that the malignant progress of BC cells is influenced by the cross talk between microRNAs (miRNAs) and ER-α signaling. However, the mechanism and functional linkage between estrogen and miRNAs remain unclear.

**Methods:**

The expression levels of miR-196a and SPRED1 in BC were tested by qRT-PCR in 46 paired BC and adjacent tissues and by the GEO datasets. The role of miR-196a in estrogen-induced BC development was examined by CCK-8 assay, wound healing assay, Matrigel invasion assay and tumorigenicity assay in nude mice. The binding site of ER-α in miR-196a promoter region was analyzed by ChIP-seq, ChIP assay and luciferase reporter assay. The potential targets of miR-196a in BC cells were explored using the luciferase reporter assay and western blot analysis, and the correlation between miR-196a and SPRED1 was analyzed by Spearman’s correlation analysis in BC specimens and GEO dataset. TCGA BRCA data was used to characterize the ESR1 signatures according to MSigDB gene set.

**Results:**

The expression levels of miR-196a were higher in ER-positive (ER+) breast tumors compared to ER-negative (ER-) tumor tissue samples. Besides, miR-196a was involved in estrogen-induced BC cell proliferation, migration and invasion. Notably, the up-regulation of miR-196a was mediated by a direct interaction with estrogen receptor α (ER-α) but not estrogen receptor β (ER-β) in its promoter region, and miR-196a expression levels were positively correlated to ER-α signature scores. Furthermore, SPRED1 was a new direct target of miR-196a which participated in miR-196a-promoted BC development and was suppressed by ligand-activated ER-α signal pathway. Finally, forced expression of miR-196a induced tumor growth of MCF7 cells, while inhibition of miR-196a significantly suppressed the tumor progress in vivo.

**Conclusions:**

Overall, the identification of estrogen/miR-196a/SPRED1 cascade will shed light on new molecular mechanism of estrogen signaling in BC development and therapy.

**Electronic supplementary material:**

The online version of this article (10.1186/s12943-018-0830-0) contains supplementary material, which is available to authorized users.

## Background

Breast cancer (BC) is the most common cancer in women worldwide, and 70% of BC is estrogen receptor α (ER-α) positive [1, 2]. Estrogen plays an important role in BC development and progression, mostly through binding and activating nuclear transcription factors (ER-α and ER-β) to induce or repress gene expression [3–5]. Previous studies documented that ER-α, a major subtype of ER, has great potential to be involved in the control of BC cell proliferation, invasion and apoptosis [6, 7]. Classically, ER-α is thought to function as a ligand-activated transcription factor which modulates gene expression and estrogen-induced biological functions by interacting with estrogen response elements in the promoter region of target genes. ER-α-positive BC patients can be treated by hormonal therapy such as tamoxifen, but the acquired resistance becomes one of the major obstacles in clinical treatment [8]. However, the underlying mechanisms of acquired resistance to hormonal therapy have not been fully elucidated, which has already become a difficult challenge to BC therapy. Therefore, it is critical to further understand new molecular mechanisms of ER-α in mediating estrogen-induced tumor progression to develop potential new therapeutic strategies in BC.

MiRNAs are small (18–23 nucleotides), endogenous noncoding RNAs that post-transcriptionally regulate gene expression by targeting the 3′-untranslated region (3′-UTR) of mRNAs [9, 10]. Aberrant expression levels of miRNAs have been reported to be associated with malignant phenotype in various types of cancers including BC [11, 12]. Recently, accumulating evidence showed that except for protein-coding genes, ER regulates the expression levels of miRNAs in response to its ligand estrogen [13, 14]. It is reported that estradiol (E2), a common estrogen, regulates miR-155 expression in an ER-α dependent manner in MCF-7 cells, which represses TP53INP1, cleaved-caspase-3, − 8, − 9, and p21 expression [15]. Meanwhile, some miRNAs are suppressed by E2, and might be involved in the tumor response to hormonal therapy [16]. Our previous studies showed that the expression levels of miR-196a were elevated upon E2 treatment in ER positive BC cells, indicating the involvement of miR-196a in estrogen-induced BC development [17]. It has been reported that miR-196a is significantly up-regulated in BC to regulate cell growth by targeting UBE2C [18] as an oncomiR [19]. Recent study also indicates that miR-196a is correlated with estrogen [20], however, the role and underlying mechanism of estrogen/miR-196a signaling in regulating BC progression remains to be further elucidated.

Sprouty-related EVH1 domain-containing protein 1 (SPRED1) which contains three functional domains EVH1 (N-terminal Enabled/VASP homology1), SPR (Sprouty-related) and KBD (C-Kit binding domain), is widely known as a tumor suppressor. Expression levels of SPRED1 were demonstrated to be down-regulated in hepatocellular carcinoma (HCC) and overexpression of SPRED1 inhibited HCC proliferation, migration and invasion [21]. They further identified that SPRED1 acted as a physiological inhibitor of Ras/Raf-1/ERK pathway and reduced the expression levels of the secretion of matrix metalloproteinase-9 (MMP-9) and MMP-2. SPRED1 was demonstrated to be expressed specifically in the luminal epithelial cells of breast ducts, and the expression levels were reduced consistently in BC. Furthermore, the down-regulation of SPRED1 promoted the proliferation of BC [22]. However, little is known about the correlation and regulation between SPRED1 and estrogen signal pathway.

In this study, we identified miR-196a as an estrogen-induced miRNA in an ER-α dependent manner in BC. Meanwhile, we discovered that SPRED1 was one of direct targets of miR-196a which is also responsive to ER-a signaling. Herein we address several important questions: i) what is(are) the change(s) of miR-196a expression upon estrogen treatment in BC cells; ii) what is underlying mechanism of the estrogen-regulated miR-196a expression in ER+ BC cells; iii) what is/are functional target(s) of the miRNA-196a that may be associated with estrogen-induced BC progression. The answers to these questions would provide new insights into a better understanding of the role of estrogen/miR-196a signal in BC development, which is helpful to provide a potential therapeutic strategy to overcome resistance to hormonal treatment in the future.

## Methods

### Human tissue samples

Human BC samples and adjacent normal tissues were obtained from patients in the First affiliated hospital of Nanjing Medical University. Tissue samples were collected at surgery, immediately frozen in liquid nitrogen and stored until total RNAs and proteins were extracted. All experiments were approved by the ethics committee of Nanjing Medical University.

### Cell culture and reagents

Human breast cancer cell lines (MCF7 and MDA-MB-231) were cultured in Dulbecco’s modified Eagle’s medium (DMEM; Gibco) supplemented with 10% fetal bovine serum (FBS; Gibco), 100 U/mL penicillin and 100 ng/mL streptomycin. Human embryonic kidney 293 T (HEK-293 T) cells were maintained in DMEM supplemented with 10% FBS, penicillin (100 U/ml), streptomycin (100 ng/ml) and glutamine (2 mmol/mL). All cell lines were maintained in a 37 °C incubator with 5% CO_2_. Prior to the treatment with estradiol (17β-estradiol, E2; Sigma-Aldrich Ltd.), ethanol solvent (Eth, used as negative control), cells were maintained for 3 days in DMEM without phenol red (Gibco) supplemented with 10% double charcoal–stripped FBS (Gibco) and pen/strep at 37 °C with 5% CO_2_. On the day of treatment, media were switched to DMEM without phenol red (Gibco) supplemented with 10% FBS (Gibco), 100 U/mL penicillin and 100 μg/mL streptomycin. Antibodies against SPRED1 and phosphorylated (p-) ERK1/2 were purchased from Cell Signaling Technology (Danvers, MA, USA). Antibodies against GAPDH and c-Raf were from Bioworld Technology (Atlanta, Georgia 30,305, USA).

### Lentiviral packaging and stable cell line establishment

Lentiviruses carrying miR-196a, miRNA-negative control (miR-NC), miR-196a inhibitor (anti-miR-196a) or miRNA inhibitor-negative control (anti-miR-NC) were packaged using lentiviral packaging kit in HEK-293 T cells following the manufacturer’s instructions (Open Biosystems, AL, USA). The lentiviral vector carrying miR-196a or miR-NC has red fluorescent protein (RFP) tag and the lentiviral vector carrying miR-196a inhibitor or miR-NC inhibitor has red fluorescent protein (RFP) tag, which can be used to check the efficiency of packaging using microscope. MCF7 or MDA-MB-231 cells were transduced by lentiviral soup and selected by puromycin to establish stable cell lines [23].

### Isolation of RNA, reverse transcription PCR and quantitative real-time PCR analysis

Total RNAs were extracted from cultured cells or human tissues with TRIzol reagent according to the manufacturer’s instruction (Invitrogen). The RNAs were reversely transcribed using the PrimeScript RT Reagent Kit (Takara, Japan). The housekeeping gene GAPDH was used as an internal control. The primers were as follows: SPRED1 forward primer, 5’-GAGGGAGTGGACTAAGCAGC-3′; SPRED1 reverse primer, 5′-CCTCTATCAAAAGCCCTAGCATC-3′; GAPDH forward primer, 5′-CCACCCATGGCAAATTCCATGGCA-3′; GAPDH reverse primer, 5′-TCTAGACGGCAGGTCAGGTCCACC -3′. To measure the expression levels of miR-196a, the stem-loop specific primer method was used as previously described [24, 25]. Quantitative reverse transcriptase (qRT) PCR primers were the following: miR-196a RT primer, 5′- GTCAGAAGGAATGATGCACAGCCAACAACA-3′; miR-196a PCR primers, sense: 5′-ACCTGCGTAGGTAGTTTCATGT-3′; antisense: 5′-CGTCAGAAGGAATGATGCACAG-3′. U6 RT primer: 5′-AACGCTTCACGAATTTGCGT-3′; U6 PCR primers sense: 5′-CTCGCTTCGGCAGCACA-3′; antisense: 5′-TGGTGTCGTGGAGTCG-3′. Quantitative RT-PCR was performed using SYBR Premix Dimer Eraser (Vazyme Biotech co.,ltd, China) on a 7900HT system. GAPDH or U6 levels were used as an internal control, and fold changes were calculated by relative quantification (2^–ΔΔCt^).

### Luciferase reporter assay

The 3′-UTR-luciferase reporter constructs containing the 3′-UTR region of SPRED1 with the wild-type and mutant binding sites of miR-196a were amplified using PCR method. The PCR products were cloned into the pmirGLO vector (Promega) between SacI and XhoI sites, immediately downstream of the luciferase gene. The mutant 3′-UTR constructs were made by introducing four mismatch mutations into the putative seed regions of SPRED1. All the constructs containing 3′-UTR region inserts were sequenced and validated. 293 T cells (1.0 × 10^5^/well) were seeded in 24-well plates. After 24 h, cells were co-transfected with either wild-type (WT) or mutant-type (mut) luciferase reporter plasmid, and equal amounts of miR-196a or miR-NC using Lipofectamine 2000 (Invitrogen) according the manufacturer’s instruction. Luciferase activities were measured 24 h after transfection using the Dual Luciferase Reporter Assay System (Promega). Experiments were performed in triplicate with three independent replicates.

The pGL3-luciferase reporters were cloned as above with wild type (WT) or mutant (mut) miR-196a promoter region in the MCS. MCF7 and MDA-MB-231 cells were pretreated with estrogen free medium and were seeded in 24-well plates. After 24 h, cells were co-transfected with either wild-type (WT) or mutant-type (mut) luciferase reporter plasmid, pRL-TK plasmid, and were treated with 10 nM E2 or equal volume Eth. Luciferase activities were measured 24 h after transfection.

### Protein extraction and immunoblotting

Cells or tissues grounded in liquid nitrogen were lysed on ice for 30 min in radioimmunoprecipitation assay (RIPA) buffer (150 mM NaCl, 100 mM Tris, pH 8.0, 0.1% sodium dodecyl sulfate (SDS), 1% Triton X-100, 1% sodium deoxycholate, 5 mM EDTA, and 10 mM NaF) supplemented with 1 mM sodium vanadate, 2 mM aprotinin, 2 mM leupeptin, 1 mM phenylmethylsulfonyl fluoride, 1 mM dithiothreitol, and 2 mM pepstatin A. The lysates were centrifugated at 12,000 rpm at 4 °C for 15 min, the supernatants were collected, and protein concentrations were determined using bicinchoninic acid assay. Protein extracts were separated by SDS–polyacrylamide gel electrophoresis and transferred to nitrocellulose membranes in transfer buffer (20 mM Tris, 150 mM glycine, 20% [volume/volume] methanol). Membranes were blocked with 5% nonfat dry milk for 2 h, then incubated with primary antibodies (SPRED1, Abcam, Cambridge, MA, USA; GAPDH, Proteintech Technology, Chicago, IL, USA; c-Raf, Cell Signaling Technology, Inc., Danvers, MA, USA; p-ERK1/2, Cell Signaling Technology, Inc., Danvers, MA, USA). The protein bands were probed with secondary antibody, and visualized with the electrochemiluminescence detection system (Thermo Scientific, Rockford, IL, USA).

### Cell proliferation assay

Cells in the logarithmic phase of growth were seeded at 3000/well, and cultured in 96-well plates. Cell proliferation was assayed using the Cell-Counting Kit 8 (CCK8; Dojindo Laboratories) according to the manufacturer’s instructions at the indicated time points. Three independent experiments were performed in triplicate [26].

### Wound healing assay

Cells were cultured until they reach 90% confluence in 6-well plates. Cell layers were scratched using a 10 μL tip to form wounded gaps, washed with PBS twice and cultured. The wounded gaps were photographed at different time points and analyzed by measuring the distance of migrating cells from five different areas for each wound [27].

### Invasion assays

Cell invasion was determined using 24-well invasion chambers with Matrigel (Becton Dickinson) according to the manufacturer’s instruction. Cells (5 × 10^4^/well) were seeded in the upper well of the invasion chamber in DMEM without serum. The lower chamber well contained DMEM supplemented with 10% FBS to stimulate cell invasion. After incubation for 24 h, non-invaded cells were removed from the top well with a cotton swab, while the bottom cells were fixed with 3% paraformaldehyde, stained with 0.1% crystal violet, and photographed in 3 independent 10× fields for each well. Membrane was air-dried and soaked for 15 min at room temperature with 33% acetic acid decolorization (200 μL/well). The destained solution was transferred to 96-well plates, and the absorbance value was read at 570 nm. Three independent experiments were conducted in triplicate [28].

### GSEA

The BRCA TCGA dataset was downloaded and re-analyzed to get miRNAs, mRNAs and clinical data for each BC patient. The dataset was split into two groups (hsa-miR-196a high expression group and hsa-miR-196a low expression group) based on the expression levels of miR-196a, and the GSEA program was employed to analyze the enrichment of each group to the gene sets DOANE BREAST CANCER CLASSES UP and DOANE BREAST CANCER ESR1 DN [29, 30].

### ChIP-seq re-analysis

The Chip-seq primary data (GSE25021) were downloaded from GEO database. This dataset is to analyze the change of the binding enriched binding abilities of ER-α with miR-196a in its promoter region using ER-α antibody for chromatin immunoprecipitation with E2 or Eth treatment. The SRA data were changed to fastq format by sratoolkit.2.6.3 software and analyzed the quality by fastqc software. The fastq format data were map to the genome bowtie2 software, after which were call peaks by MAC2 software. The results were visualized by IGV.

### ESR1 signatures

The ESR1 signatures were analyzed using the gene set (C2 CGP; DOANE_BREAST_CANCER_CLASSES_UP) form Molecular Signatures Database in MSigDB. TCGA BRCA data were downloaded and were analyzed the ESR1 signatures by the methods described before [31, 32].

### ChIP assay

The sequence of miR-196a promoter was obtained from the UCSC Genome Database. Analysis of putative transcription factor binding sites on hsa-miR-196a-1 promoter was performed using JASPAR. The ChIP assay was performed using the ChIP Assay Kit (Beyotime, China). MCF7 and MDA-MB-231 cells treated with E2 or ethanol, then lysed using SDS lysis buffer. Total DNAs in the cells were sheared by sonication. Protein-DNA complexes were precipitated by control immunoglobulin G and anti-ER-α antibody respectively, followed by eluting the complex from the antibody. PCR analysis was carried out with primers specific for miR-196a.

### Tumorigenesis in nude mice

Male nude mice (BALB/c-null, 6-week-old) were purchased from Shanghai Laboratory Animal Center (Chinese Academy of Sciences, Shanghai, China), and bred in special pathogen-free (SPF) condition. All studies were approved by the Institutional Committee on Animal Care of Nanjing Medical University. For tumor growth assay, MCF7 cells stably expressing miR-196a, miR-NC, anti-miR-196a or anti-miR-NC were injected subcutaneously into both flanks of nude mice (8 mice each group, 5 × 10^6^ cells in 100 μl serum-free DMEM medium). Tumor sizes were measured using vernier caliper every two days when the tumors were visible. Tumor volumes were calculated according to the formula: volume = 0.5 × Length×Width^2^. Mice were sacrificed 17 days after implantation, and tumors were dissected. Total proteins were extracted for Western blot analysis to test specific protein expression levels. Tumors were formalin-fixed, paraffin-embedded, sectioned at 5 μm and stained using CD31 (Abcam) and Ki67 (Abcam) antibody under the standard procedure as we previously described [33].

### Statistical analysis

Data in the present study were represented as means ± SD from at least three independent experiments except those specifically indicated. Student’s unpaired *t* test was used for comparison between two groups. Data were analyzed using GraphPad Prism 5 (La Jolla). For human tissue samples, SPRED1 expression levels in adjacent normal and BC tissues were explored by Student’s paired *t* test. SPRED1 levels in ERα-positive and negative groups were analyzed using Student’s unpaired *t* test. Values were considered significantly different at *P <* 0.05.

## Results

### MiR-196a is up-regulated in human BC, especially in ER+ tumor tissues

**Fig. 1:**
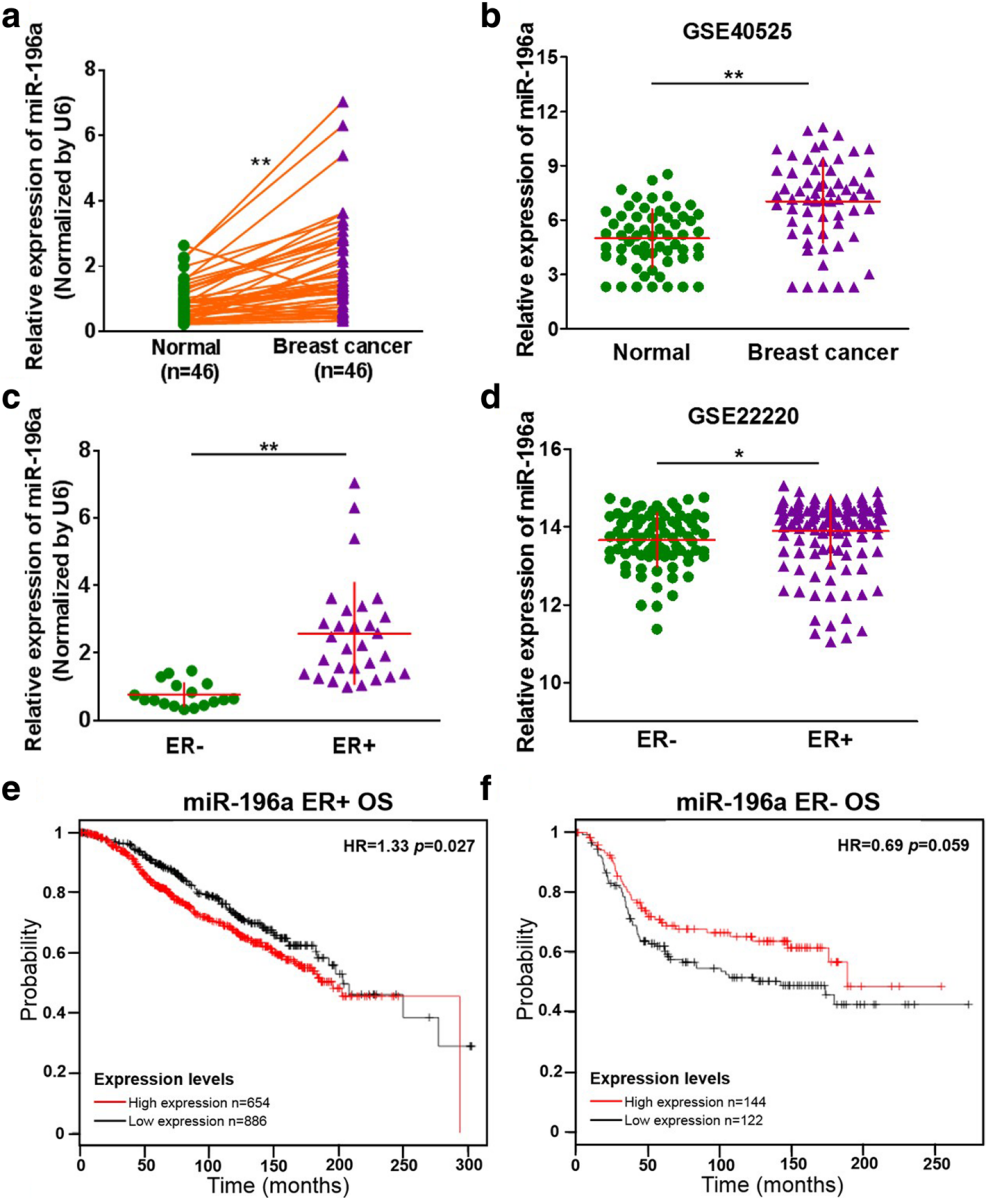
MiR-196a is up-regulated in human BC, especially in ER+ tumor tissues. **a** The expression levels of miR-196a in 46 paired of BC and adjacent normal tissues were analyzed by qRT-PCR and normalized to U6 expression levels. Student’s *t*-test was used to analyze the difference between the non-tumor tissues and BC group. ** indicates significant difference at *P <* 0.01. **b** The miR-196a expression levels of normal adjacent breast tissues and BC tissues were analyzed in the BC database of the public GEO dataset (GSE40525). ** indicates significant difference at *P <* 0.01. **c** The relative miR-196a expression levels of BC tumors were analyzed according to ER status (ER-negative, *n* = 17; ER-positive, *n* = 29). Data were presented as mean from three independent experiments with triple replicates per experiment. ** indicates significant difference at *P <* 0.01. **d** Different GEO dataset GSE22220 was used to analysis the expression levels of miR-196a in ER-negative or ER-positive tissues. * indicates significant difference at *P <* 0.05. **e**, **f** The Kaplan Meier plotter was used to detect the overall survival (OS) of miR-196a in ER+ and ER- BC patients, respectively

High levels of miR-196a have been reported in various solid cancers including BC [18, 34]. However, there is still lack of study about the differential expression of miR-196a in ER+ and ER- BC tumor tissues. Quantitative reverse-transcriptase PCR (qRT-PCR) assay showed that in 46 pairs of human breast tissue samples, the expression levels of miR-196a in BC tumor tissues were significantly higher than those in the adjacent normal tissues (Fig. 1a). Similarly, the independent BC gene expression data sets (GSE2669) from public database GEO showed that miR-196a expression levels were significantly up-regulated in BC tissues (Fig. 1b). Next, we analyzed the expression levels of miR-196a in our ER+ and ER- BC specimens, and the results demonstrated that miR-196a expression levels were significantly higher in ER+ BC tissues than those in ER- group (Fig. 1c). Meanwhile, analysis of the GEO datasets, a database repository of high throughput gene expression data containing miRNA expression profiling for cohorts of ER– and ER+ breast cancers, also showed the similar results (Fig. 1d, Additional file 1: Figure S1). In addition, high expression levels of miR-196a indicated poor OS prognosis in ER+ BC patients, but not in ER- BC patients which implicated importance of miR-196a in ER+ BC (Fig. 1e and f). These results demonstrate that miR-196a expression levels are correlated with not only BC malignancy, but also ER status of tumors, indicating that miR-196a may be regulated by estrogen receptor in BC development.

### Silence of miR-196a reverses the tumor-promoting effects of E2 in ER+ BC cells

**Fig. 2:**
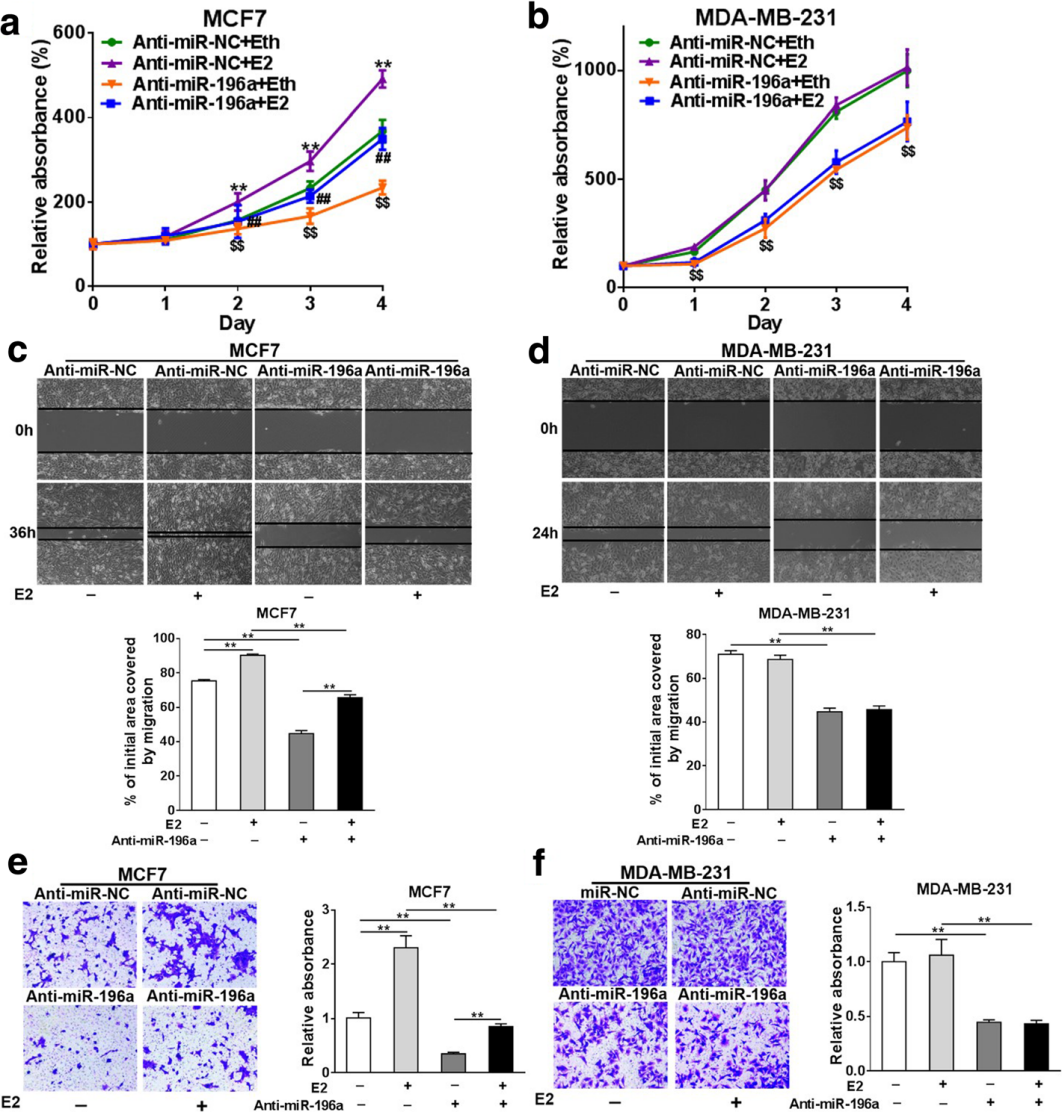
Silence of miR-196a reverses the tumor-promoting effects of E2 in ER+ BC cells. ER+ BC cells MCF7 and ER- BC cells MDA-MB-231 were cultured with estrogen-free medium for 72 h before treatment. The cells were transfected with the inhibitor (Anti-miR-196a) or control anti-sense RNA inhibitor (Anti-miR-NC). **a**, **b** These cells were seeded at 3000 cells/well in 96-well plates, then treated with 10 nM estradiol (E2) or ethyl alcohol (Eth). Cell Counting Kit-8 (CCK-8) Kit was used to detect cell vitality every 24 h. Data were presented as the means± SD from three independent experiments. ** indicates significant difference between Anti-miR-NC with E2 treatment (Anti-miR-196a + Eth) group and Anti-miR-NC without E2 treatment (Anti-miR-NC + Eth) group. $$ indicates significant difference between Anti-miR-196a + Eth group and Anti-miR-NC + Eth group. ## indicates significant difference between Anti-miR-196a + E2 group and Anti-miR-NC + E2 group. **c**, **d** The cells above were used to perform wound healing assay to analyze the ability of cell migration. ** indicates significant difference between indicated groups. **e**, **f** The cells above were used to perform Matrigel invasion assay using 10 mice per treatment. ** indicated significant difference between indicated groups

As widely reported, estrogens stimulate the proliferation and metastatic potential of BC cells. In our study,we also observed that E2 treatment increased tumor growth in ER+ MCF7 BC cells, but not in ER- MDA-MB-231 cells (Additional file 2: Figure S2 A-D). To evaluate the role of miR-196a in estrogen (E2)-mediated BC development, we first determined whether E2-regulated miR-196 affects BC development. MCF7 and MDA-MB-231 cells were transfected with anti-miR-196a inhibitor or anti-miR-NC, then treated with or without E2. Although anti-miR-196a inhibitor reduced cell proliferation in both MCF7 and MDA-MB-231 cells without E2 stimulation, the anti-miR-196a inhibitor reversed the E2-promoted cell proliferation of only the ER+ BC cells MCF7, but not of ER- BC cells MDA-MB-231 (Fig. 2a and b). Similarly, interference of miR-196a attenuated E2-induced migration and invasion in MCF7 cells, but not in MDA-MB-231 cells (Fig. 2c-f). These results indicate that miR-196a is required for E2-induced ER+ BC progression such as cell proliferation, migration and invasion.

### miR-196a expression is induced by E2, and miR-196a is a gene signature associated with ER-α

**Fig. 3:**
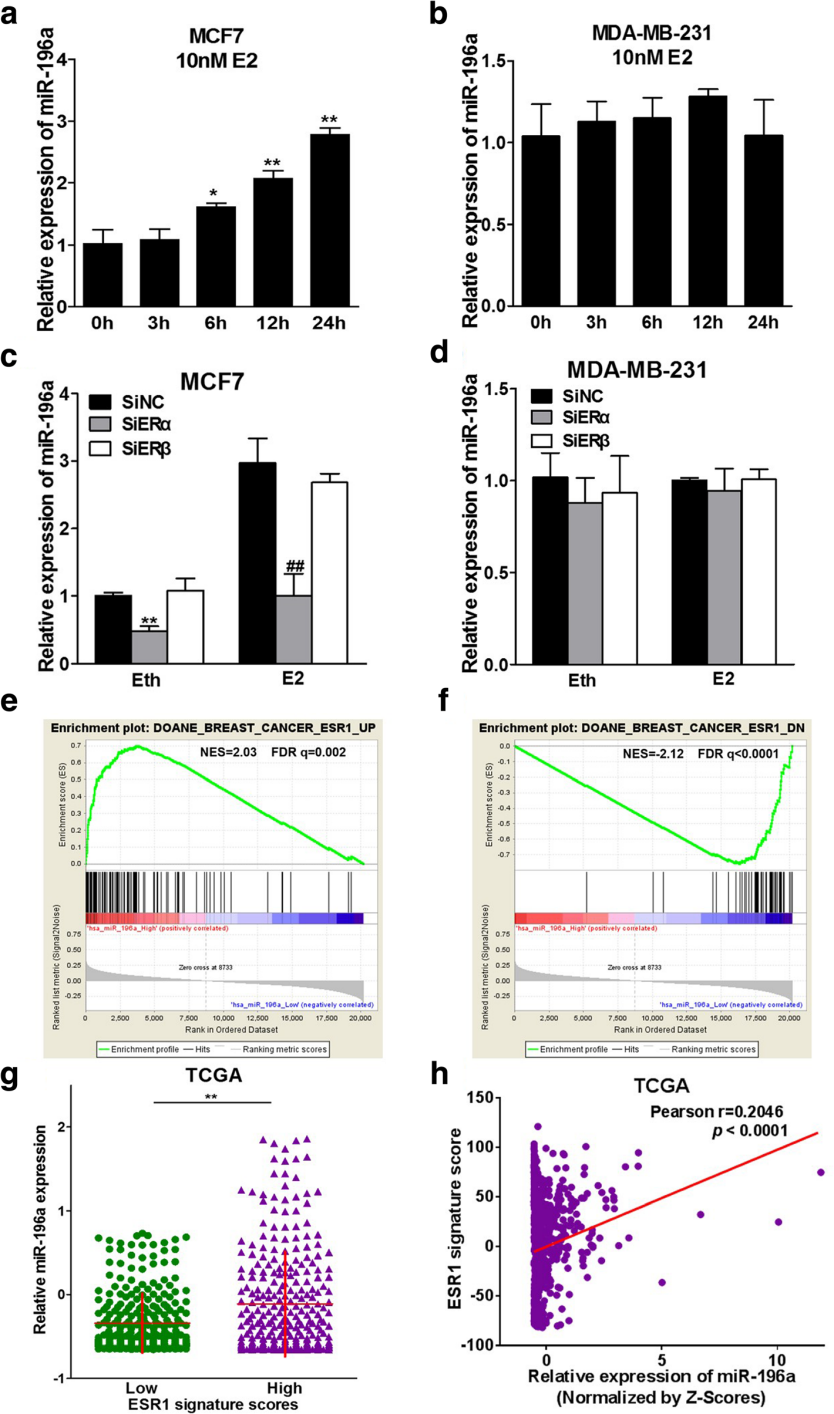
miR-196a expression is induced by E2 and miR-196a is a gene signature associated to ER-α. **a**, **b** ER+ BC cells MCF7 and ER- BC cells MDA-MB-231 were cultured with estrogen-free medium for 72 h, then treated with 10 nM E2 or equal amount of solvent Eth for 0, 3, 6, 12 or 24 h. The expression levels of miR-196a were analyzed by qRT-PCR and normalized to the values of the Eth control. U6 levels were used as an internal control. Data were presented as means ± SD from three independent experiments with triple replicates per experiment. * and ** indicate significant difference compared to the 0 h group at *P <* 0.05 and *P <* 0.01, respectively. **c**, **d** Cells were treated as above, then transfected with 50 nM ERα siRNAs, ERβ siRNAs or negative control siRNAs (siNC). After 24 h, the cells were treated with 10 nM E2 or Eth, the expression levels of miR-196a were analyzed by qRT-PCR and U6 levels were used an internal control, and normalized to the value of siNC+Eth group. ** indicates significant difference between the siNC+Eth group and the siERα+Eth group at *P <* 0.01. ## indicates significant difference between the siNC+E2 and the siERα+E2 group and at *P <* 0.01. **e**, **f** GSEA program were employed to analyze the ERα signal enrichment scores between miR-196a high and low expression group using the TCGA BRCA dataset. **g** Primary human breast cancers of TCGA BRCA dataset were classified to high or low ERα signature score group, and the expression levels of miR-196a were analyzed in the two groups. The details were described in methods and materials. ** indicates significant difference between ERα signature high score and low score group. **h** Pearson’s correlation analysis was used to determine the correlation between the expression levels of ERα (ESR1 signature score) and miR-196a expression levels

Previous studies have shown that estrogen is an important factor in BC by regulating gene expression including miRNAs, and our results also indicate the ER signal pathway participates in the abnormal expression of miR-196a in ER+ BC cells. To explore the relationship between ER signal pathway and miR-196a, we further investigated whether estrogen was involved in regulation of miR-196a levels. The results showed that E2 markedly induced miR-196a expression in a time-dependent manner in MCF7 cells, but not in MDA-MB-231 cells (Fig. 3a and b). We also observed the expression levels of miR-196b, the other member of miR-196 family, showed no significant change (Additional file 3: Figure S3). It has been shown that estrogen mediates its effects by binding to its receptors. To further confirm whether the effect of estrogen is through its receptors ER-α and/or ER-β, we found that E2 treatment promoted miR-196a expression. ER-α, but not ER-β knockdown significantly decreased miR-196a expression only in MCF7 cells with or without E2 treatment, indicating that ER-α, but not ER-β, is necessary for E2-induced miR-196a expression in ER+ BC cells (Fig. 3c and d; Additional file 4: Figure S4 A and B). To evaluate the relationship between miR-196a and ER-α expression levels, we downloaded BRCA TCGA mRNAs and miRNAs expression dataset and divided the samples into miR-196a high and miR-196a low expression groups. ER-α is encoded by ESR1 gene. We analyzed ESR1 signal enrichment scores in each group using GSEA program. The results showed that miR-196a high expression group was enriched in DOANE BREAST CANCER ESR1 UP dataset (NES = 2.03, FDR q = 0.002), whereas miR-196a low expression group was enriched in DOANE BREAST CANCER ESR1 DOWN dataset (NES = − 2.12, FDR q < 0.001), confirming the positive correlation between miR-196a and ER-α expression levels (Fig. 3e and f). In addition, we calculated the ESR1 signature scores of BRCA TCGA samples in DOANE BREAST CANCER ESR1 UP dataset and divided the samples into high and low ER-α signature scores groups. We then analyzed the expression levels of miR-196a in the two groups, and the results showed that the expression levels of miR-196a in high ER-α signature scores group was more than those in low ER-α signature scores group (Fig. 3g). Furthermore, we tested the Pearson’s correlation between miR-196a expression levels and ER-α signature scores. In addition, we found that the expression levels of miR-196a in the ER+ tumors with the E2 treatment were higher than in the Eth group, however, there were no significant difference in ER- tumors (Additional file 2: Figure S2 E and F). The results showed that miR-196a expression levels were positively correlated to ESR1 signature scores, and there was a positive correlation between miR-196a and ER-α expression levels (Fig. 3h; Additional file 5: Figure S5). Overall, these results indicate that miR-196a expression levels were induced by E2 in ER+ BC cells and were closely correlated with ER-α signaling.

### Estrogen promotes the binding of ER-α in the miR-196a promoter region in ER+ BC cells

**Fig. 4:**
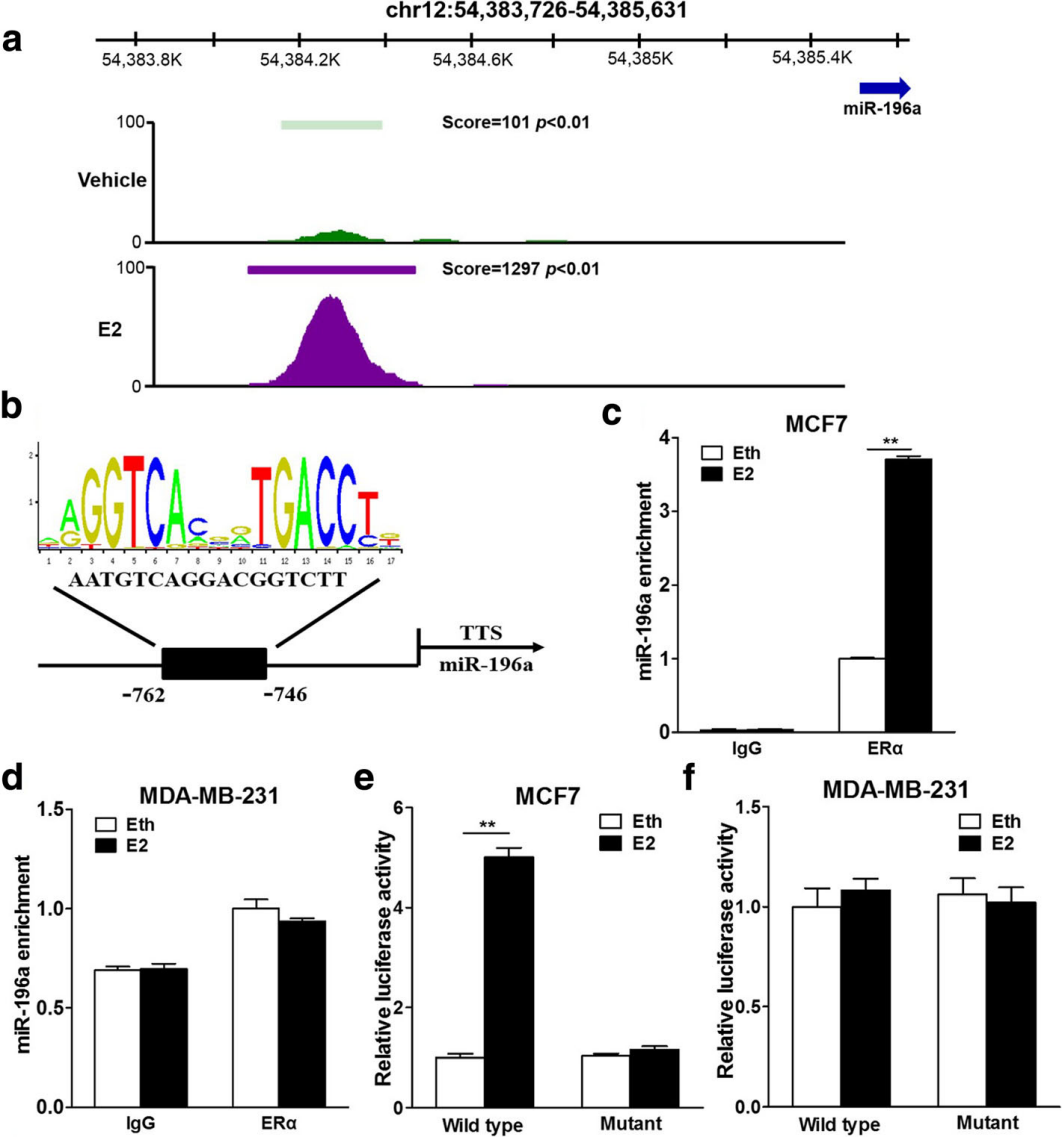
Estrogen promotes the binding of ER-α in the miR-196a promoter region in ER+ BC cells. **a** GEO dataset GSE25021 was reanalyzed to identify the binding sites and enrichment intensity of ERα in the promoter region of miR-196a, and the call peaks results were displayed by IGV. **b** The JASPAR program was employed to seek the seed binding sites of ERα in the promoter region of miR-196a. **c**, **d** The MCF7 and MDA-MB-231 cells were cultured with estrogen-free medium for 72 h t, then treated with 10 nM E2 or equal amount of solvent ethyl alcohol (Eth) for 24 h. The ChIP assay was performed to determine the binding sites of ERα in the promoter region of miR-196a. The enrichment of miR-196a in cell lysates pulled down by ERα antibody was analyzed by qRT-PCR and normalized to the value of ERα + Eth group. ** indicates significant difference at *P <* 0.01. **e**, **f** The wild type (AATGTCAGGACGGTCTT) or mutant (ACTCTAAGGACCGCCCT) seed binding site of miR-196a promoter region was subcloned into pGL3 luciferase reporter vector and verified by sequencing. The cells were pretreated as above, transfected with the wild type or mutant reporter construct, pRL-TK plasmid, and treated with Eth or E2. After 24 h, the dual-luciferase assay was performed to analyze the luciferase activities of each group. ** indicates significant difference at *P <* 0.01

It has long been known that ER-α is considered to act as a ligand-activated transcription factor, but its function remains elusive. As the data shown above, miR-196a expression levels were mediated by ER-α signal pathway. Therefore, we further analyzed whether ER-α transcriptionally regulates miR-196a expression by binding to the promoter region of miR-196a. First, analysis of the primary ChIP-seq data (GSE25021) from GEO database using MCF7 cells with E2 and vehicle control (ethanol, Eth) treatment showed that there was significant ER-α enrichment signal in the promotor region of miR-196a upon E2 treatment, indicating that the induction of miR-196a by E2 might be through the enriched binding abilities of ER-α in the miR-196a promoter region (Fig. 4a). Next, we downloaded the sequence from the binding peak and predicted the potential seed binding sequence of ER-α by transcription factor binding profile database JASPAR, and there was indeed seed region matching the ER-α binding model in miR-196a promoter region (Fig. 4b). To validate the enrichment of ER-α in the promoter region of miR-196a induced by E2, we performed ChIP assay to analyze the binding enrichment of ER-α in the seed region with or without E2 treatment in MCF7 and MDA-MB-231 cells. As we expected, the enrichment of the ER-α in miR-196a upstream was only significantly upregulated upon E2 treatment in ER+ BC cells (Fig. 4c and d). Furthermore, to evaluate whether the ER-α binding site plays biological roles in transcriptional activation of miR-196a response to E2 stimulation, we constructed the pGL3 luciferase reporter vectors containing wild type or mutant type with mutation of seed sequence of the ER-α binding site in miR-196a promoter region, then tested luciferase activities of BC cells with or without E2 treatment. The results showed that E2 promoted the transcriptional activity of miR-196a only in MCF7 cells, but not in MDA-MB-231 cells (Fig. 4e and f). These results demonstrate that estrogen induces miR-196a transcriptional activation by promoting the binding of ER-α in miR-196a promoter region in ER+ BC cells.

### MiR-196a directly targets and inhibits SPRED1

**Fig. 5:**
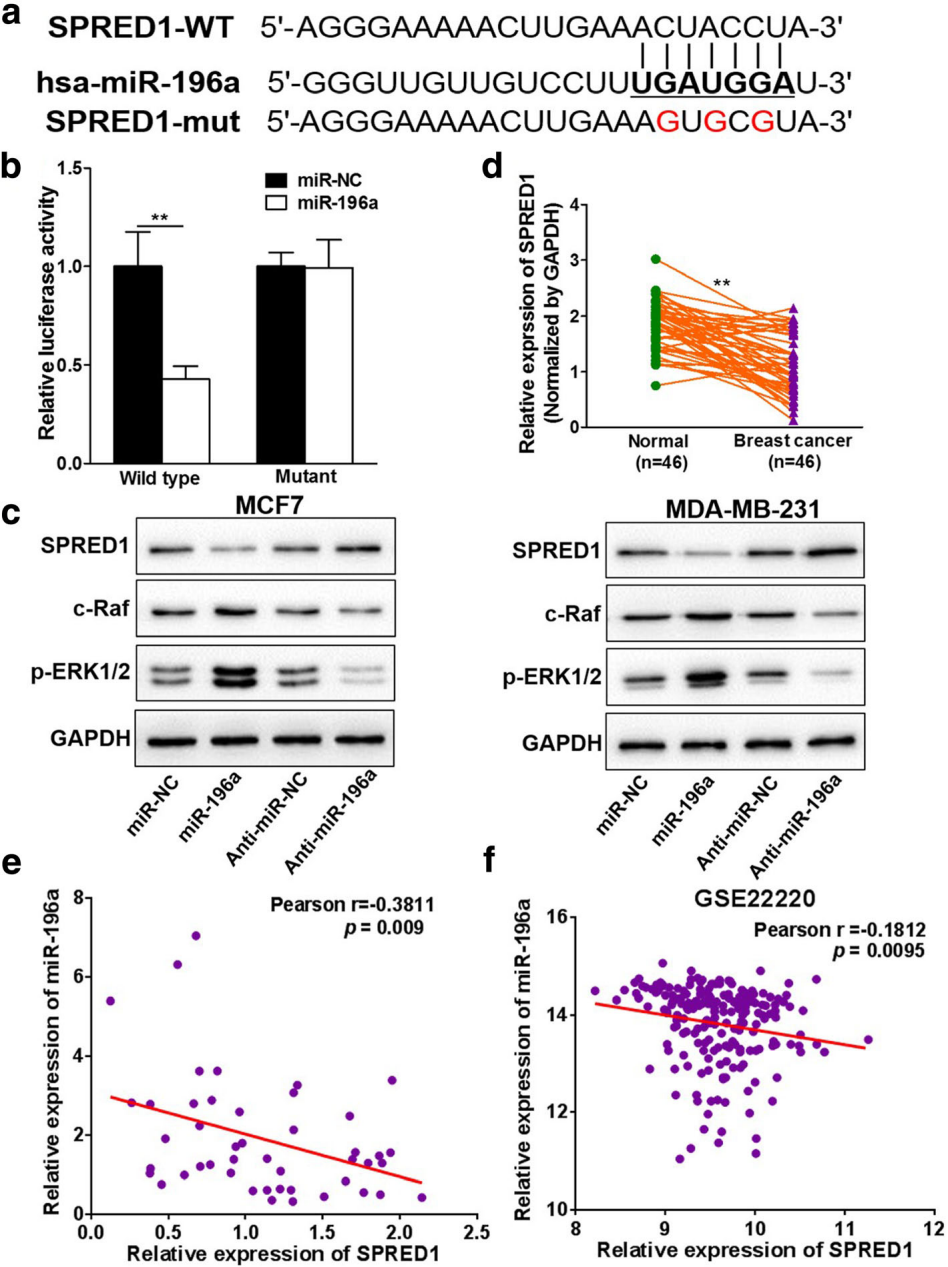
MiR-196a directly targets and inhibits SPRED1. **a** Putative seed-matching sites (in bold) or mutant sites (red) between miR-196a and 3’-UTR of SPRED1 were analyzed by TargetScan, miRWalk 2.0 and miRBase. **b** The wild type (WT) and mutant (mut) SPRED1 3′-UTR reporter constructs were made and verified by sequencing. Luciferase reporter assay was performed using 293 T cells to detect the relative luciferase activities of WT and mut SPRED1 reporters. Renilla luciferase vector was used as an internal control. Data were presented as the means ± SD from three independent experiments with quadruple replicates per experiment. ** indicates significant difference compared to the control at *P <* 0.01. **c** Protein expression levels of SPRED1, c-Raf, pERK1/2 and GAPDH were determined using Western blot analysis in MCF7 and MDA-MB-231 cells overexpressing miR-196a, miR-NC or anti-miR-196a inhibitor and anti-miR-NC. **d** The expression levels of SPRED1 in adjacent normal tissues and human BC specimens were determined by qRT-PCR, and the fold changes were obtained by the ratio of SPRED1 to GAPDH levels. ** indicates significant difference compared to the normal tissues *P <* 0.01. **e** Pearson’s correlation analysis was used to determine the correlation between the expression levels of SRED1 and miR-196a in human BC specimens (*n* = 46). **f** GEO dataset GSE22220 was used to analyze the Pearson’s correlation between miR-196a and SPRED1 levels

To further understand molecular mechanisms by which miR-196a exhibits its biological effects on BC cells, several computational methods (TargetScan, miRWalk 2.0, miRBase) were used to identify potential miR-196a targets. Among the candidates, we found that seed sequence of miR-196a matched 3′-UTR region of SPRED1 (Fig. 5a). To test whether miR-196a targets SPRED1, SPRED1 3′-UTR containing this seed sequence was cloned into pmirGLO vector (WT). Overexpression of miR-196a in HEK-293 T cells decreased luciferase activities of wild type reporter to 45%, suggesting that miR-196a inhibits its 3′-UTR reporter activities of SPRED1. To test whether miR-196a specifically inhibits the activity by binding its seed sequence, we also made mutant construct with the mutation of miR-196a-binding site in the 3′-UTR of SPRED1 (Mut). Forced expression of miR-196a did not affect the transcriptional activation of mutant SPRED1 3′-UTR reporter activity (Fig. 5b), indicating that miR-196a directly targets SPRED1 by binding to its seed sequence at 3’-UTR. In addition, overexpression of miR-196a in MCF7 and MDA-MB-231 cells significantly suppressed SPRED1 protein expression levels, whereas anti-miR-196a inhibitor increased SPRED1 expression, demonstrating that SPRED1 is a direct target of miR-196a in BC cells (Fig. 5c). SPRED1 is considered to act as a negative regulator of the RAS/RAF/MAPK signaling [35, 36], we found that consistent with decreased SPRED1 expression, miR-196a overexpressing cells had high c-Raf and p-ERK1/2 levels, whereas anti-miR-196a inhibitor treatment significantly decreased c-Raf and p-ERK1/2 levels (Fig. 5c, Additional file 6: Figure S6). These results indicate that miR-196a could activate the RAS/RAF/MAPK signaling through targeting SPRED1.

To further identify the relationship between miR-196a and SPRED1 expression, we analyzed the expression levels of SPRED1 in human BC specimens by qRT-PCR and in the GEO dataset GSE22220. The results showed that compared to the paired adjacent normal tissues, BC tissues (*n* = 46) showed significantly lower levels of SPRED1 (Fig. 5d). Meanwhile, Pearson’s rank correlation analysis showed an inverse correlation between the expression levels of SPRED1 and miR-196a in human BC specimens (Pearson’s correlation *r* = − 0.3811, Fig. 5e). We also used the publicly available datasets GEO dataset GSE22220 to validate our results (Pearson’s correlation *r* = − 0.1812, Fig. 5f). Finally, in order to address whether overexpression of SPRED1 inhibits miR-196a-inducing malignant phenotypes miR-196a overexpressing cells were transfected with pCMV expression vector containing SPRED1 cDNA without 3′-UTR region or vector alone as negative control and used to investigate cell proliferation, migration and invasion. The results showed that overexpression of SPRED1 can restore the miR-196a-promoted cell proliferation, migration and invasion in BC cells (Additional file 7: Figure S7 A-H). Collectively, these results suggest that miR-196a/SPRED1 signaling was involved in regulating BC development through MAPK signaling, especially in ER-positive BC.

### SPRED1 is down-regulated by E2 and negatively correlated to ER status in BC cells

**Fig. 6:**
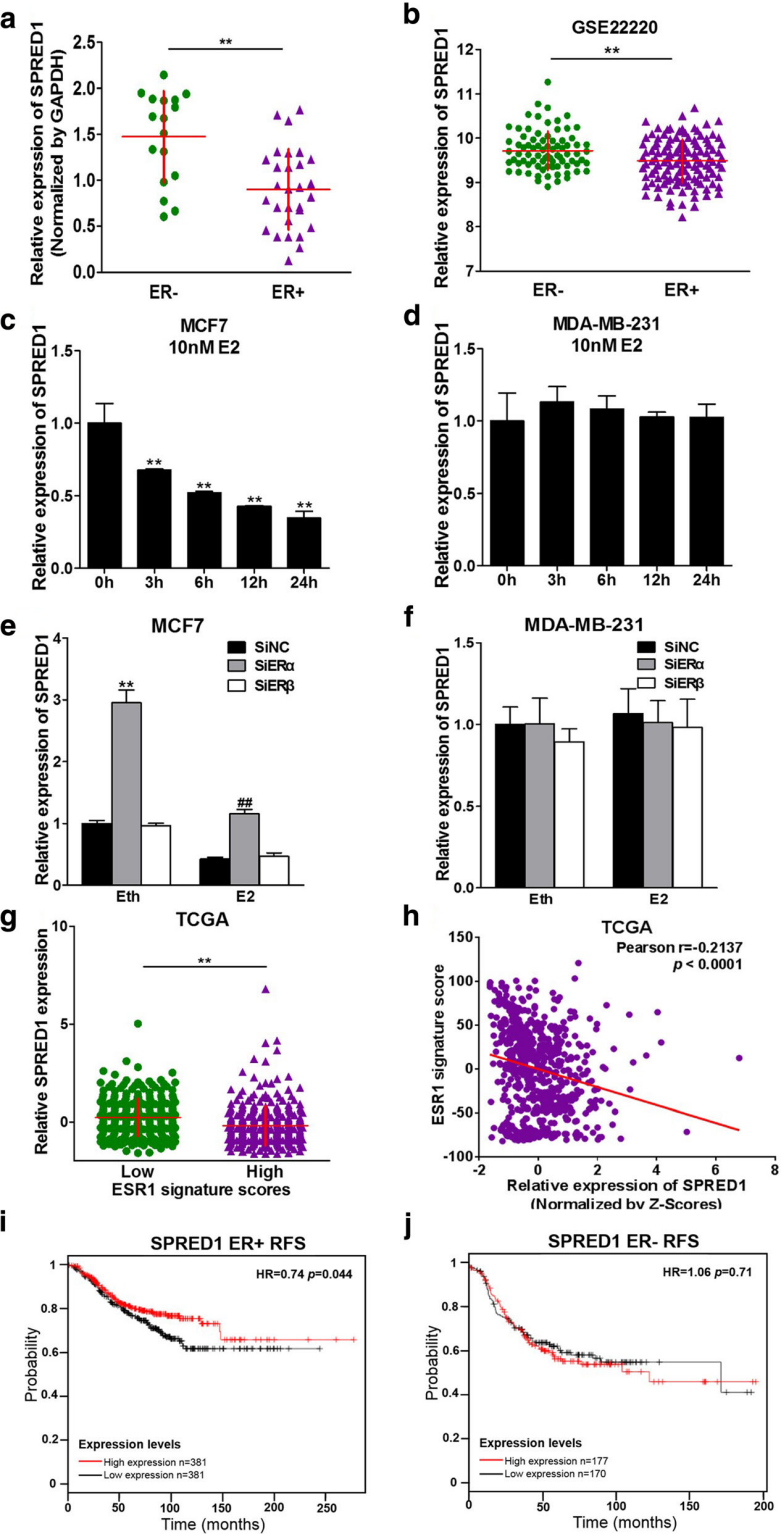
SPRED1 is down-regulated by E2 and negatively correlated to ER status in BC cells. **a** The expression levels of SPRED1 in 46 ER+ and ER- BC tissues were analyzed by qRT-PCR and normalized to GAPDH expression levels. ** indicates significant difference compared to normal tissues at *P <* 0.01. **b** GEO2R dataset GSE22220 was used to analyze the expression levels of SPRED1 in ER-negative or ER-positive tissues. ** indicates significant difference between ER-negative and ER-positive tumors at *P <* 0.01. **c**, **d** ER+ BC cells MCF7 and ER- BC cells MDA-MB-231 were cultured with estrogen-free medium for 72 h, then treated with 10 nM E2 or equal amount of solvent Eth. The expression levels of SPRED1 were analyzed using qRT-PCR, and normalized to the values of the Eth control. Data were presented as the means ± SD from three independent experiments with triple replicates per experiment. ** indicates significant difference compared to the 0 h group at *P <* 0.01. **e**, **f** Cells were treated as above, then transfected with 50 nM ERα siRNAs, ERβ siRNAs or negative control siRNAs (siNC). After 24 h, the cells were treated with 10 nM E2 or Eth, the expression levels of SPRED1 were analyzed using qRT-PCR, and normalized to the value of siNC+Eth group. ** indicates significant difference between siNC+Eth and siERα+Eth group at *P <* 0.01. ## indicates significant difference between siNC+E2 and siERα+E2 group at *P <* 0.01. **g** Primary human breast cancers of TCGA BRCA dataset were classified to high or low ESR1 signature score group, and the expression levels of SPRED1 were analyzed in these two groups. The details were described in methods. ** indicates significant difference between low ESR1 signature score group and high ESR1 signature group. **h** Pearson’s correlation analysis was used to determine the correlation between the expression levels of ESR1 signature score and SPRED1 expression levels. **i**, **j** The Kaplan Meier plotter was used to detect the relapse free survival (RFS) of SPRED1 in ER+ and ER- BC patients, respectively

The results above showed that miR-196a expression levels were induced by E2 in ER+ BC cells and SPRED1 was the direct target of miR-196a. Therefore, we further analyzed whether SRPED1 was inducted by ER signal pathway. First, we tested the expression levels of SPRED1 according to ER status in clinical BC samples, and found out that SPRED1 expression levels were significant down-regulated in ER+ specimens compared to ER- specimens in both our BC samples and GEO dataset GSE22220 (Fig. 6a and b). Furthermore, E2 treatment decreased SPRED1 expression in MCF7 cells in a time-dependent manner, whereas E2 did not change SPRED1 levels in MDA-MB-231 cells (Fig. 6c and d). However, we demonstrated that there were no changes of the other reported targets of miR-196a such as HOXA5, HOXA7, HOXB8, HOXC8, HOXD8, ANXA1, NME4 and ZEB1 upon E2 treatment (Additional file 8: Figure S8 A and B). In addition, we knocked down the expression levels of ER-α or ER-β using siRNAs and tested SPRED1 expression with or without E2 treatment. The results illuminated that SPRED1 expression levels were decreased upon E2 treatment, and ER-α, but not ER-β inhibition dramatically reversed SPRED1 levels regardless the presence of E2 in ER+ BC cells (Fig. 6e and f). We also observed that SPRED1 expression levels were down-regulated in ESR1 high signature scores group in TCGA dataset (Fig. 6g) and there was a negative correlation between ESR1 signature score and SPRED1 expression levels (Fig. 6h, Additional file 9: Figure S9). Consistently, low expression levels of SPRED1 indicated poor prognosis of ER + BC patients instead of ER- patients (Fig. 6i and j). Besides, results of tumorigenesis in nude mice showed that E2 repressed the expression levels of SPRED1 only in ER+ BC cells, while SPRED1 was suppressed by overexpression of miR-196a in both MCF7 and MDA-MB-231 cells (Additional file 2: Figure S2G and H). In summary, our results demonstrate that ER+ BC shows lower expression levels of SPRED1, and E2 treatment down-regulates SPRED1 expression, which is mediated by ER-α in ER+ BC cells.

### MiR-196a induces tumor growth in vivo

**Fig. 7:**
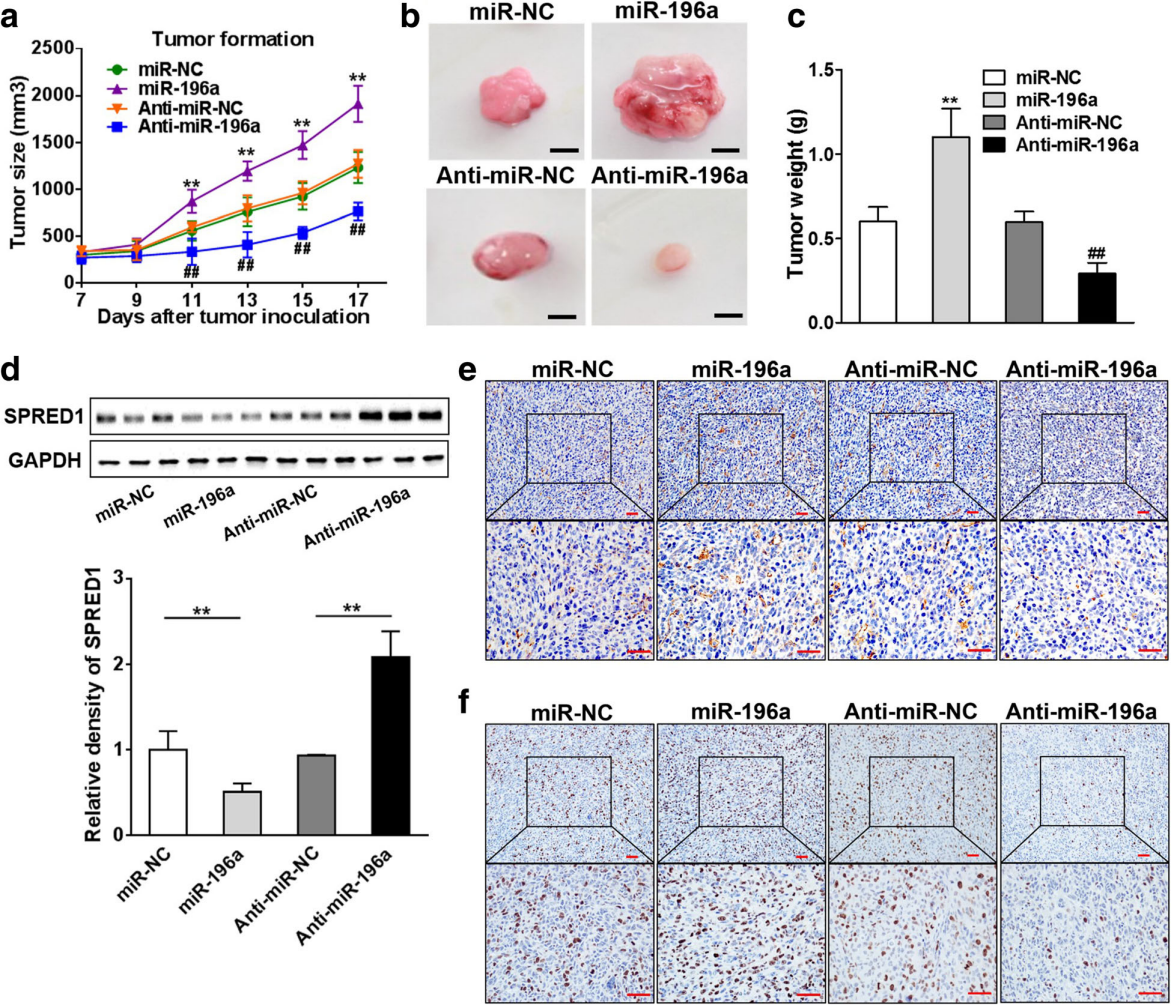
MiR-196a induces tumor growth in vivo. **a** MCF7/miR-196a, MCF7/miR-NC, MCF7/anti-miR-196a, or MCF7/anti-miR-NC cells (5 × 10^6^cells) were dispersed in 100 μl of serum-free DMEM medium and subcutaneously injected into the both sides of posterior flank of female nude mice (*n* = 8). Tumors growth was measured every two days after the tumors were detected in 7 days and the tumor volumes were calculated using the following formula: volume = 0.5 × Length × Width^2^. ** indicates significant difference compared to miR-NC group at *P <* 0.01; ## indicates significant difference compared to anti-miR-NC group at *P <* 0.01. **b** The representative pictures of trimmed tumors (Bar = 10 mm). **c** The tumors were excised and weighed after 17 days. Data were presented as the means± SD from all tumors. ** indicates significant difference compared to miR-NC group at *P <* 0.01. ## indicates significant difference compared to Anti-miR-NC group at *P <* 0.01. **d** The total proteins were extracted from xenografts and subjected to Western blot analysis for SPRED1 expression. GAPDH expression level was served as an internal control. **e**, **f** The expression of CD31 and Ki67 were analyzed in tumor tissues by immunohistochemistry (bar = 50 μm). ** indicates significant difference compared to the miR-NC group at *P <* 0.01; ## indicates significant difference compared to the anti-miR-NC group at *P <* 0.01

In order to further investigate the effect of miR-196a on BC tumorigenesis, MCF7 cells were infected with lentivirus carrying miR-196a or anti-miR-196a inhibitor, followed by the selection of puromycin. Stable cell lines expressing miR-NC or miR-NC inhibitor (anti-miR-NC) were also established as controls. Cells were injected subcutaneously into both flanks of nude mice, and tumor sizes were measured after one week. Compared with control group, the overexpression of miR-196a significantly promoted tumor growth, whereas miR-196a inhibitor markedly attenuated tumor growth (Fig. 7a). After 17 days, the xenografts were trimmed out and weighed. As shown in Fig. 7b and c, miR-196a overexpression significantly increased tumor size and weight, which were inhibited by anti-miR-196a inhibitor compared to anti-miR-NC group. Furthermore, the protein levels of SPRED1 in xenografts from miR-196a-expressing cells were much lower than those from miR-NC control cells, confirming the negative correlation between miR-196a and SPRED1 in vivo (Fig. 7d). Moreover, the tumors from miR-196a-overexpressing MCF7 cells showed increased number of CD31 positive microvessels and higher expression levels of Ki67 by IHC staining, whereas the tumors from anti-miR-196a inhibitor group showed decreased microvessel densities and Ki67 expression levels compared to controls (Fig. 7e and f; Additional file 10: Figure S10). These results suggest that ER-regulated miR-196a enhances tumor growth and angiogenesis which is associated with its reduced expression of target SPRED1 in vivo.

## Discussion

Estrogen plays an important role in the development and progression of BC, more than two-thirds of breast tumors express ER that are prone to exhibit resistance to endocrine therapy [37, 38]. In the present study, we are interested in role and mechanism of ER–regulated miR-196a and its target in BC development. Although miR-196a has been reported to be involved in various types cancers including head and neck cancer, gastric cancer, ovarian carcinoma and BC [39–42]; there is no information about the underlying mechanisms of estrogen-regulated miR-196a in BC progression. We demonstrated that miR-196a expression levels were higher in the ER+ BC tissues compared to ER- tissues, which was consistent with the results from GSE22220 dataset showing the noteworthy correlation between miR-196a levels and ER status in BC. Some reports have shown that miR-196a participates in the development of BC and acts as an oncogene [18, 34, 43]. Similarly, we demonstrated that interference of miR-196a attenuated estrogen-induced cell proliferation, migration and invasion in ER+ BC cells, demonstrating an important role of miR-196a in estrogen-promoted BC development. These results indicate that the aberrant expression of miR-196a is correlated to ER signal pathway in BC progression, and miR-196a is potential target in hormone mediated BC development.

Estrogen and miRNA correlation has been explored both for predictive biomarker of development and therapeutic target identification [44, 45]. It was reported that the tumor suppressor Let-7 targets ER-α [46, 47]. Several reports have demonstrated that estrogen regulates microRNA processing and expression at post-transcriptional level [48, 49]. For example, miR-760 and miR-424 were down-regulated, whereas miR-618, miR-570, and miR-107 were up-regulated upon the E2 stimulation [50]. Estrogen mediated the activation of miR-191/425 cluster in ER positive BC cells [51]. Our results demonstrated that miR-196a was significantly increased with the E2 treatment in ER-positive cell line MCF7, but not in ER-negative cell line MDA-MB-231. ER is composed of two subunits ER-α and ERβ, which contain three functional domains: NH2-terminal domain (NTD); DNA-binding domain (DBD); COOH-terminal ligand-binding domain (LBD), nevertheless, they share little common sequence. Classically, ER-α is thought to interact with estrogen response elements (EREs) in the promoter regions of the regulated genes and act as a ligand-activated transcription factor, and mediate the transcription of the target genes [52]. It has been reported that ER-β also has extensive roles in mediating gene expression including equilibrating ER-α signal pathway, but its function remains elusive [53]. Our results showed that ER-α, but not ER-β, was involved in mediating miR-196a expression by ER activation, which was a novel mechanism of regulation. To better understand the mechanism of estrogen-induced miR-196a up-regulation, we re-analyzed the ChIP-seq data from GEO database containing MCF7 ChIP assay results with or without E2 treatment using ER-α antibody. The results elucidated that there was more enrichment in the promoter region of miR-196a by ER-α upon E2 stimulation compared to Eth treatment as solvent control. We further identified the transcriptional regulation of ER-α upon E2 treatment in the promoter region of miR-196a using ChIP assay and promoter reporter assay in MCF7 and MDA-MB-231 cells. The results validated that ER+ MCF7 cells, but not ER- MDA-MB-231 cells, showed stronger affinity of ER-α with the ERE in miR-196a promoter region upon E2 treatment when compared to Eth control. We also observed the positive correlation between miR-196a and ER-α expression levels using GSEA assay showing that miR-196a expression levels were positively correlated to ER-α signatures scores and ER-α expression levels in BC specimens. Additionally, we found that ER-α responsive miR-196a still expressed in ER- breast cancer cells, which is independent of ER signal pathway and consistent to previous studies [54, 55]. However, the mechanism of the miR-196a regulation in ER- BC cells remain unknown which would need further study. Thus, we demonstrated that E2 promoted the binding of ER-α to the specific ERE sites in the promoter of miR-196a to regulate its expression in ER+ BC cell.

Previous reports have demonstrated several miR-196 targets, including HOX family (A5/A7/B8/C8/D8), AnnexinA1, NME4, ZEB1 [56] and we found there were no significant changes of these miR-196 targets upon E2 treatment in MCF7 cells. To identify the direct target(s) of miR-196a regulated by E2, we used the bioinformatic programs and verified that SPRED1 was a novel target of E2-promoted miR-196a. SPRED1 is a member of the sprouty and sprouty-related protein family as a negative regulator of growth factor signaling [57]. Meanwhile, SPRED1 functions by forming a complex with Raf and inhibits activation of ERK pathway [21]. SPRED1 also suppresses cell proliferation and migration in various tumors [21, 58]. In our study, we confirmed the negative correlation between miR-196a and SPRED1 expression levels in both BC cells and BC tumor tissues. We further verified that SPRED1 was a direct target of miR-196 and it played pivotal roles in miR-196a promoted BC cell proliferation, migration and invasion by inhibiting c-Raf signal pathway. Furthermore, we found that SPRED1 expression was negatively correlative to estrogen signal pathway, and ER-α recovered SPRED1 expression in ER+ BC cells. In addition, we also analyzed the expression levels of several miRNAs that were reported to be involved in regulation of SPRED1, and found that E2 stimulation did not significantly change their expression levels. Consequently, our results suggest that miR-196a and its target SPRED1 play an important role in estrogen-mediated BC progression, which may be helpful to provide new therapeutic target for ER+ BC in the future.

Overall, our findings show the novel role and mechanism of estrogen/miR-196a/SPRED1 signaling in the estrogen-induced BC development. These new results may provide potential clinical application for BC diagnosis or as new therapeutic target(s) in the future.

## Conclusions

Here, we identified that miR-196a was up-regulated by estrogen in ER positive BC cells and enriched in ER-positive BC tissues. Moreover, estrogen-induced miR-196a was positively correlated with ER-α signature scores and promoted BC development in vitro and in vivo by targeting SPRED1. Furthermore, we demonstrated that miR-196a up-regulation by estrogen was through ERα, but not ERβ. ERα directly bound to the promotor of miR-196a. Thus, our study provides a novel mechanism of estrogen-induced BC development, which may be used as new biomarkers for the diagnosis and treatment of ER-positive BC in the future.

## Additional files

Additional file 1: **Figure S1.** MiR-196a is up-regulated in ER+ BC tissues. (A, B) Two different GEO2R datasets GSE58215 and GSE40267 were used to analysis the expression levels of miR-196a in ER-negative or ER-positive tissues. * and ** indicate significant difference compared to the 0 h group at *P* < 0.05 and *P* < 0.01, respectively. (JPEG 88 kb)

Additional file 2: **Figure S2.** Estrogen induces tumor growth of ER+ but ER- BC cells via the regulation of miR-196a. The MCF7 and MDA-MB-231 miR-NC- and miR-196a-overexpressing cells (2 × 10^6^cells) were dispersed in 100 μl of serum-free DMEM medium, and subcutaneously injected into both sides of posterior flank of nude mice. The mice were fed with E2 (0.67 μg/ml) or Eth contained water since the tumors were detected in 7 days (6 mice each group). (A-D) The tumors were excised and weighed after 17 days, and the representative pictures of trimmed tumors were displayed (Bar = 10 mm). Data were presented as the means± SD from all tumor samples. ** indicates significant difference at *P* < 0.01. (E-H) The expression levels miR-196a and SPRED1 from the tumors were analyzed by qRT-PCR and normalized to the values of the Eth + miR-NC group. ** indicates significant difference at *P* < 0.01. (JPEG 444 kb)

Additional file 3: **Figure S3.** MiR-196b has no response to E2 treatment in ER+ BC cells. (A) ER+ BC cells MCF7 and ER- BC cells MDA-MB-231 were cultured with estrogen-free medium for 72 h before E2 treatment, then treated with 10 nM E2 or equal amount of solvent Eth as solvent control for 0, 3, 6, 12 or 24 h. The expression levels of miR-196b were analyzed by qRT-PCR and U6 levels were used as internal control, and normalized to the values of the Eth control. Data were presented as the means ± SD from three independent experiments with triple replicates per experiment. * and ** indicate significant difference compared to the 0 h group at *P* < 0.05 and *P* < 0.01, respectively. (JPEG 37 kb)

Additional file 4: **Figure S4.** The interference effects of siERα and siERβ in BC cells. (A, B) MCF7 and MDA-MB-231 cells were transfected with 50 nM ERα siRNAs, ERβ siRNAs or negative control siRNAs (siNC), respectively. After 48 h, the expression levels of ESR1 or ESR2 were analyzed by qRT-PCR and GAPDH levels were used as an internal control, and normalized to the value of siNC group in each cell line, respectively. ** indicates significant difference compared to the siNC group at *P* < 0.01. (JPEG 73 kb)

Additional file 5: **Figure S5.** miR-196a is positively associated with ESR1 expression levels. (A) Pearson’s correlation analysis was used to determine the correlation between the expression levels of ERα and miR-196a expression levels. (PNG 58 kb)

Additional file 6: **Figure S6.** MiR-196a directly targets and inhibits SPRED1. (A-F) Protein expression levels of SPRED1, c-Raf, pERK1/2 and GAPDH were determined using Western blot analysis in MCF7 and MDA-MB-231 cells overexpressing miR-196a, miR-NC or anti-miR-196a inhibitor and anti-miR-NC. The data were normalized as the ratio of miR-NC group, respectively. **indicated significant difference between indicated groups at *P* < 0.01. (JPEG 189 kb)

Additional file 7: **Figure S7.** SPRED1 reverses miR-196a-induced malignant phenotype of BC cells. MiR-196a- or miR-NC-overexpressing MCF7 cells were transfected with vector or SPRED1 cDNA without 3’-UTR. (A-B) The expression levels of SPRED1, c-Raf, pERK1/2 and GAPDH were determined by Western blot analysis after 48 h of transfection. (C-D) Cell viability was detected using CCK-8 assay. (E-F) Cells were treated and wound healing assay was performed as above. (G-H) Transwell invasion assay was performed as above using control cells and cells overexpressing miR-196a with or without SPRED1 overexpression. Data were presented as the means ± SD from three independent experiments with triple replicates per experiment. * and ** indicate significant difference between the miR-NC + Vector group and the miR-196a + Vector group with *P* < 0.05 and *P* < 0.01, respectively. # and ## indicate significant difference between the miR-196a + Vector group and the miR-196a + SPRED1 group with *P* < 0.05 and *P* < 0.01, respectively. (JPEG 552 kb)

Additional file 8: **Figure S8.** The typical targets of miR-196a shows no change with E2 treatment in ER+ BC cells. (A) The MCF7 cells were cultured with estrogen-free medium for 72 h before E2 treatment, then treated with 10 nM E2. After 24 h, the expression levels of some reported targets of miR-196a were analyzed by qRT-PCR. (B) ER+ BC cells MCF7 were cultured with estrogen-free medium for 72 h, then treated with 10 nM E2 or equal amount of solvent Eth for 24 h. The expression levels of miRNAs which were reported to be involved in regulation of SPRED1 were analyzed by qRT-PCR and normalized to the values of the Eth control. (JPEG 105 kb)

Additional file 9: **Figure S9.** The correlation between SPRED1 and ESR1 expression levels. (A) Pearson’s correlation analysis was used to determine the correlation between the expression levels of ERα and SPRED1 expression levels. (JPEG 62 kb)

Additional file 10: **Figure S10.** The densities of CD31 levels were analyzed in tumor tissues with changes of miR-196a. MCF7/miR-196a, MCF7/miR-NC, MCF7/anti-miR-196a, or MCF7/anti-miR-NC cells were dispersed in 100 μl of serum-free DMEM medium and subcutaneously injected into the sides of posterior flank of nude mice (*n* = 4). The tumors were excised and sent to H&E (bar = 1000 μm) and immunohistochemistry to analyze the expression levels of CD31after 17 days. The densities of CD31 levels were quantified by ImageJ software, and presented as the means ± SD from 8 tumor tissues. (TIFF 1043 kb)

## Abbreviations

3′-UTR: 3′-untranslated regions
BC: Breast cancer
BS: Binding site
ChIP: Chromatin Immunoprecipitation
ChIP-Seq: Chromatin Immunoprecipitation Sequencing
E2: Estradiol
ER: Estrogen receptor
ERE: Estrogen response element
ERα: Estrogen receptor α
ERβ: Estrogen receptor β
GSEA: Gene Set Enrichment Analysis
miR-196a: microRNA-196a
miRNAs: microRNAs
qRT-PCR: quantitative real-time PCR
SPRED1: Sprouty-related EVH1 domain-containing protein 1

## References

[1] Ban KA, Godellas CV (2014). Epidemiology of breast cancer. Surg Oncol Clin N Am.

[2] Bolt MJ, Stossi F, Callison AM, Mancini MG, Dandekar R, Mancini MA (2015). Systems level-based RNAi screening by high content analysis identifies UBR5 as a regulator of estrogen receptor-alpha protein levels and activity. Oncogene.

[3] Chan HJ, Li H, Liu Z, Yuan YC, Mortimer J, Chen S (2015). SERPINA1 is a direct estrogen receptor target gene and a predictor of survival in breast cancer patients. Oncotarget.

[4] Thomas C, Gustafsson JA (2011). The different roles of ER subtypes in cancer biology and therapy. Nat Rev Cancer.

[5] Nilsson S, Gustafsson JA (2011). Estrogen receptors: therapies targeted to receptor subtypes. Clin Pharmacol Ther.

[6] Maruani DM, Spiegel TN, Harris EN, Shachter AS, Unger HA, Herrero-Gonzalez S, Holz MK (2012). Estrogenic regulation of S6K1 expression creates a positive regulatory loop in control of breast cancer cell proliferation. Oncogene.

[7] Cao P, Feng F, Dong G, Yu C, Feng S, Song E, Shi G, Liang Y, Liang G (2015). Estrogen receptor alpha enhances the transcriptional activity of ETS-1 and promotes the proliferation, migration and invasion of neuroblastoma cell in a ligand dependent manner. BMC Cancer.

[8] Molloy ME, White BE, Gherezghiher T, Michalsen BT, Xiong R, Patel H, Zhao H, Maximov PY, Jordan VC, Thatcher GR, Tonetti DA (2014). Novel selective estrogen mimics for the treatment of tamoxifen-resistant breast cancer. Mol Cancer Ther.

[9] Lewis BP, Shih IH, Jones-Rhoades MW, Bartel DP, Burge CB (2003). Prediction of mammalian microRNA targets. Cell.

[10] He L, Hannon GJ (2004). MicroRNAs: small RNAs with a big role in gene regulation. Nat Rev Genet.

[11] Lin S, Gregory RI (2015). MicroRNA biogenesis pathways in cancer. Nat Rev Cancer.

[12] Lee JM, Cho KW, Kim EJ, Tang Q, Kim KS, Tickle C, Jung HS (2015). A contrasting function for miR-137 in embryonic mammogenesis and adult breast carcinogenesis. Oncotarget.

[13] Yu X, Zhang X, Dhakal IB, Beggs M, Kadlubar S, Luo D (2012). Induction of cell proliferation and survival genes by estradiol-repressed microRNAs in breast cancer cells. BMC Cancer.

[14] Bhat-Nakshatri P, Wang G, Collins NR, Thomson MJ, Geistlinger TR, Carroll JS, Brown M, Hammond S, Srour EF, Liu Y, Nakshatri H (2009). Estradiol-regulated microRNAs control estradiol response in breast cancer cells. Nucleic Acids Res.

[15] Zhang C, Zhao J, Deng H (2013). 17beta-estradiol up-regulates miR-155 expression and reduces TP53INP1 expression in MCF-7 breast cancer cells. Mol Cell Biochem.

[16] Maillot G, Lacroix-Triki M, Pierredon S, Gratadou L, Schmidt S, Benes V, Roche H, Dalenc F, Auboeuf D, Millevoi S, Vagner S (2009). Widespread estrogen-dependent repression of micrornas involved in breast tumor cell growth. Cancer Res.

[17] Jiang CF, Li DM, Shi ZM, Wang L, Liu MM, Ge X, Liu X, Qian YC, Wen YY, Zhen LL (2016). Estrogen regulates miRNA expression: implication of estrogen receptor and miR-124/AKT2 in tumor growth and angiogenesis. Oncotarget.

[18] Han Q, Zhou C, Liu F, Xu G, Zheng R, Zhang X (2015). MicroRNA-196a post-transcriptionally upregulates the UBE2C proto-oncogene and promotes cell proliferation in breast cancer. Oncol Rep.

[19] Lee SJ, Seo JW, Chae YS, Kim JG, Kang BW, Kim WW, Jung JH, Park HY, Jeong JY, Park JY (2014). Genetic polymorphism of miR-196a as a prognostic biomarker for early breast cancer. Anticancer Res.

[20] Kim K, Madak-Erdogan Z, Ventrella R, Katzenellenbogen BS (2013). A MicroRNA196a2* and TP63 circuit regulated by estrogen receptor-alpha and ERK2 that controls breast cancer proliferation and invasiveness properties. Horm Cancer.

[21] Yoshida T, Hisamoto T, Akiba J, Koga H, Nakamura K, Tokunaga Y, Hanada S, Kumemura H, Maeyama M, Harada M (2006). Spreds, inhibitors of the Ras/ERK signal transduction, are dysregulated in human hepatocellular carcinoma and linked to the malignant phenotype of tumors. Oncogene.

[22] Lo TL, Yusoff P, Fong CW, Guo K, McCaw BJ, Phillips WA, Yang H, Wong ES, Leong HF, Zeng Q (2004). The ras/mitogen-activated protein kinase pathway inhibitor and likely tumor suppressor proteins, sprouty 1 and sprouty 2 are deregulated in breast cancer. Cancer Res.

[23] Xu Q, Liu LZ, Yin Y, He J, Li Q, Qian X, You Y, Lu Z, Peiper SC, Shu Y, Jiang BH (2015). Regulatory circuit of PKM2/NF-kappaB/miR-148a/152-modulated tumor angiogenesis and cancer progression. Oncogene.

[24] Chen C, Ridzon DA, Broomer AJ, Zhou Z, Lee DH, Nguyen JT, Barbisin M, Xu NL, Mahuvakar VR, Andersen MR (2005). Real-time quantification of microRNAs by stem-loop RT-PCR. Nucleic Acids Res.

[25] Wang X (2009). A PCR-based platform for microRNA expression profiling studies. RNA.

[26] Xu Q, Liu LZ, Qian X, Chen Q, Jiang Y, Li D, Lai L, Jiang BH (2012). MiR-145 directly targets p70S6K1 in cancer cells to inhibit tumor growth and angiogenesis. Nucleic Acids Res.

[27] Wang L, Jiang CF, Li DM, Ge X, Shi ZM, Li CY, Liu X, Yin Y, Zhen L, Liu LZ, Jiang BH. MicroRNA-497 inhibits tumor growth and increases chemosensitivity to 5-fluorouracil treatment by targeting KSR1. Oncotarget. 2016;7(3):2660-71. 10.18632/oncotarget.6545 PMC4823062 26673620

[28] Shi ZM, Wang L, Shen H, Jiang CF, Ge X, Li DM, Wen YY, Sun HR, Pan MH, Li W (2017). Downregulation of miR-218 contributes to epithelial-mesenchymal transition and tumor metastasis in lung cancer by targeting slug/ZEB2 signaling. Oncogene.

[29] Subramanian A, Tamayo P, Mootha VK, Mukherjee S, Ebert BL, Gillette MA, Paulovich A, Pomeroy SL, Golub TR, Lander ES, Mesirov JP (2005). Gene set enrichment analysis: a knowledge-based approach for interpreting genome-wide expression profiles. Proc Natl Acad Sci U S A.

[30] Mootha VK, Lindgren CM, Eriksson KF, Subramanian A, Sihag S, Lehar J, Puigserver P, Carlsson E, Ridderstrale M, Laurila E (2003). PGC-1alpha-responsive genes involved in oxidative phosphorylation are coordinately downregulated in human diabetes. Nat Genet.

[31] Adorno M, Cordenonsi M, Montagner M, Dupont S, Wong C, Hann B, Solari A, Bobisse S, Rondina MB, Guzzardo V (2009). A mutant-p53/Smad complex opposes p63 to empower TGFbeta-induced metastasis. Cell.

[32] Enzo E, Santinon G, Pocaterra A, Aragona M, Bresolin S, Forcato M, Grifoni D, Pession A, Zanconato F, Guzzo G (2015). Aerobic glycolysis tunes YAP/TAZ transcriptional activity. EMBO J.

[33] Liu LZ, Zheng JZ, Wang XR, Jiang BH (2008). Endothelial p70 S6 kinase 1 in regulating tumor angiogenesis. Cancer Res.

[34] Yuan Y, Anbalagan D, Lee LH, Samy RP, Shanmugam MK, Kumar AP, Sethi G, Lobie PE, Lim LH (2016). ANXA1 inhibits miRNA-196a in a negative feedback loop through NF-kB and C-Myc to reduce breast cancer proliferation. Oncotarget.

[35] Pasmant E, Gilbert-Dussardier B, Petit A, de Laval B, Luscan A, Gruber A, Lapillonne H, Deswarte C, Goussard P, Laurendeau I (2015). SPRED1, a RAS MAPK pathway inhibitor that causes Legius syndrome, is a tumour suppressor downregulated in paediatric acute myeloblastic leukaemia. Oncogene.

[36] Phoenix TN, Temple S (2010). Spred1, a negative regulator of Ras-MAPK-ERK, is enriched in CNS germinal zones, dampens NSC proliferation, and maintains ventricular zone structure. Genes Dev.

[37] Deblois G, Giguere V (2013). Oestrogen-related receptors in breast cancer: control of cellular metabolism and beyond. Nat Rev Cancer.

[38] Liang J, Shang Y (2013). Estrogen and cancer. Annu Rev Physiol.

[39] Suh YE, Raulf N, Gaken J, Lawler K, Urbano TG, Bullenkamp J, Gobeil S, Huot J, Odell E, Tavassoli M (2015). MicroRNA-196a promotes an oncogenic effect in head and neck cancer cells by suppressing annexin A1 and enhancing radioresistance. Int J Cancer.

[40] Sun M, Liu XH, Li JH, Yang JS, Zhang EB, Yin DD, Liu ZL, Zhou J, Ding Y, Li SQ (2012). MiR-196a is upregulated in gastric cancer and promotes cell proliferation by downregulating p27(kip1). Mol Cancer Ther.

[41] Fan Y, Fan J, Huang L, Ye M, Huang Z, Wang Y, Li Q, Huang J (2015). Increased expression of microRNA-196a predicts poor prognosis in human ovarian carcinoma. Int J Clin Exp Pathol.

[42] Hui AB, Shi W, Boutros PC, Miller N, Pintilie M, Fyles T, McCready D, Wong D, Gerster K, Waldron L (2009). Robust global micro-RNA profiling with formalin-fixed paraffin-embedded breast cancer tissues. Lab Investig.

[43] Chen Y, Huang S, Wu B, Fang J, Zhu M, Sun L, Zhang L, Zhang Y, Sun M, Guo L, Wang S (2017). Transforming growth factor-beta1 promotes breast cancer metastasis by downregulating miR-196a-3p expression. Oncotarget.

[44] Bailey ST, Westerling T, Brown M (2015). Loss of estrogen-regulated microRNA expression increases HER2 signaling and is prognostic of poor outcome in luminal breast cancer. Cancer Res.

[45] Chu HW, Cheng CW, Chou WC, Hu LY, Wang HW, Hsiung CN, Hsu HM, Wu PE, Hou MF, Shen CY, Yu JC (2014). A novel estrogen receptor-microRNA 190a-PAR-1-pathway regulates breast cancer progression, a finding initially suggested by genome-wide analysis of loci associated with lymph-node metastasis. Hum Mol Genet.

[46] Zhao Y, Deng C, Wang J, Xiao J, Gatalica Z, Recker RR, Xiao GG (2011). Let-7 family miRNAs regulate estrogen receptor alpha signaling in estrogen receptor positive breast cancer. Breast Cancer Res Treat.

[47] Boyerinas B, Park SM, Hau A, Murmann AE, Peter ME (2010). The role of let-7 in cell differentiation and cancer. Endocr Relat Cancer.

[48] Pinho FG, Frampton AE, Nunes J, Krell J, Alshaker H, Jacob J, Pellegrino L, Roca-Alonso L, de Giorgio A, Harding V (2013). Downregulation of microRNA-515-5p by the estrogen receptor modulates sphingosine kinase 1 and breast cancer cell proliferation. Cancer Res.

[49] Guo X, Yang C, Qian X, Lei T, Li Y, Shen H, Fu L, Xu B (2013). Estrogen receptor alpha regulates ATM expression through miRNAs in breast cancer. Clin Cancer Res.

[50] Cicatiello L, Mutarelli M, Grober OM, Paris O, Ferraro L, Ravo M, Tarallo R, Luo S, Schroth GP, Seifert M (2010). Estrogen receptor alpha controls a gene network in luminal-like breast cancer cells comprising multiple transcription factors and microRNAs. Am J Pathol.

[51] Di Leva G, Piovan C, Gasparini P, Ngankeu A, Taccioli C, Briskin D, Cheung DG, Bolon B, Anderlucci L, Alder H (2013). Estrogen mediated-activation of miR-191/425 cluster modulates tumorigenicity of breast cancer cells depending on estrogen receptor status. PLoS Genet.

[52] Nassa G, Tarallo R, Guzzi PH, Ferraro L, Cirillo F, Ravo M, Nola E, Baumann M, Nyman TA, Cannataro M (2011). Comparative analysis of nuclear estrogen receptor alpha and beta interactomes in breast cancer cells. Mol BioSyst.

[53] Williams C, Edvardsson K, Lewandowski SA, Strom A, Gustafsson JA (2008). A genome-wide study of the repressive effects of estrogen receptor beta on estrogen receptor alpha signaling in breast cancer cells. Oncogene.

[54] Hoffman AE, Zheng T, Yi C, Leaderer D, Weidhaas J, Slack F, Zhang Y, Paranjape T, Zhu Y (2009). microRNA miR-196a-2 and breast cancer: a genetic and epigenetic association study and functional analysis. Cancer Res.

[55] Jin K, Sukumar S. A pivotal role for HOXB7 protein in endocrine resistant breast cancer. Oncoscience. 2015;2(11):917-9. 10.18632/oncoscience.263 PMC4675788 26697525

[56] Lu YC, Chang JT, Chan EC, Chao YK, Yeh TS, Chen JS, Cheng AJ (2016). miR-196, an emerging Cancer biomarker for digestive tract cancers. J Cancer.

[57] Inoue H, Kato R, Fukuyama S, Nonami A, Taniguchi K, Matsumoto K, Nakano T, Tsuda M, Matsumura M, Kubo M (2005). Spred-1 negatively regulates allergen-induced airway eosinophilia and hyperresponsiveness. J Exp Med.

[58] Miyoshi K, Wakioka T, Nishinakamura H, Kamio M, Yang L, Inoue M, Hasegawa M, Yonemitsu Y, Komiya S, Yoshimura A (2004). The Sprouty-related protein, Spred, inhibits cell motility, metastasis, and rho-mediated actin reorganization. Oncogene.

