# Supplementary figures and images for: Estrogen-induced miR-196a elevation promotes tumor growth and metastasis via targeting SPRED1 in breast cancer
Source: Mol Cancer. 2018 Apr 23;17:83. doi: 10.1186/s12943-018-0830-0 (PMC5914046; doi:10.1186/s12943-018-0830-0)

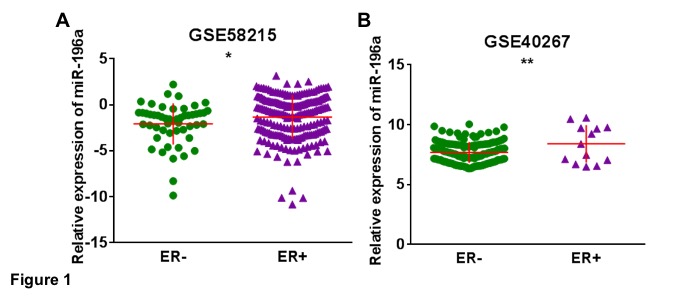

Supplement: Supplementary file 1 — Figure S1. MiR-196a is up-regulated in ER+ BC tissues. (A, B) Two different GEO2R datasets GSE58215 and GSE40267 were used to analysis the expression levels of miR-196a in ER-negative or ER-positive tissues. * and ** indicate significant difference compared to the 0 h group at P < 0.05 and P < 0.01, respectively. (JPEG 88 kb) [file 12943_2018_830_MOESM1_ESM.jpg]

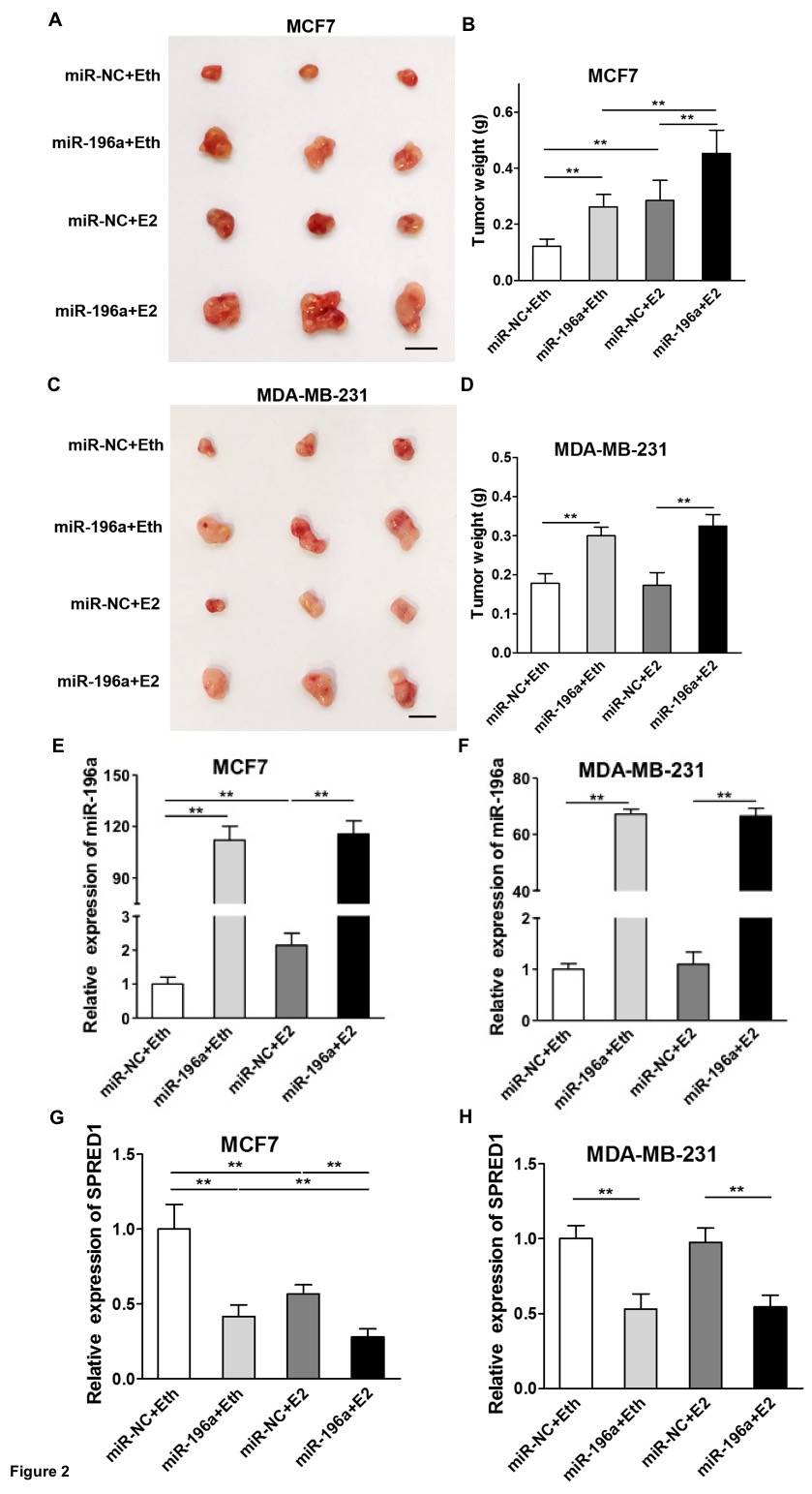

Supplement: Supplementary file 2 — Figure S2. Estrogen induces tumor growth of ER+ but ER- BC cells via the regulation of miR-196a. The MCF7 and MDA-MB-231 miR-NC- and miR-196a-overexpressing cells (2 × 10^6^cells) were dispersed in 100 μl of serum-free DMEM medium, and subcutaneously injected into both sides of posterior flank of nude mice. The mice were fed with E2 (0.67 μg/ml) or Eth contained water since the tumors were detected in 7 days (6 mice each group). (A-D) The tumors were excised and weighed after 17 days, and the representative pictures of trimmed tumors were displayed (Bar = 10 mm). Data were presented as the means± SD from all tumor samples. ** indicates significant difference at P < 0.01. (E-H) The expression levels miR-196a and SPRED1 from the tumors were analyzed by qRT-PCR and normalized to the values of the Eth + miR-NC group. ** indicates significant difference at P < 0.01. (JPEG 444 kb) [file 12943_2018_830_MOESM2_ESM.jpg]

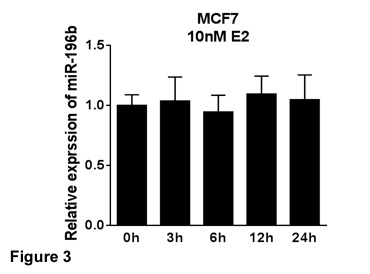

Supplement: Supplementary file 3 — Figure S3. MiR-196b has no response to E2 treatment in ER+ BC cells. (A) ER+ BC cells MCF7 and ER- BC cells MDA-MB-231 were cultured with estrogen-free medium for 72 h before E2 treatment, then treated with 10 nM E2 or equal amount of solvent Eth as solvent control for 0, 3, 6, 12 or 24 h. The expression levels of miR-196b were analyzed by qRT-PCR and U6 levels were used as internal control, and normalized to the values of the Eth control. Data were presented as the means ± SD from three independent experiments with triple replicates per experiment. * and ** indicate significant difference compared to the 0 h group at P < 0.05 and P < 0.01, respectively. (JPEG 37 kb) [file 12943_2018_830_MOESM3_ESM.jpg]

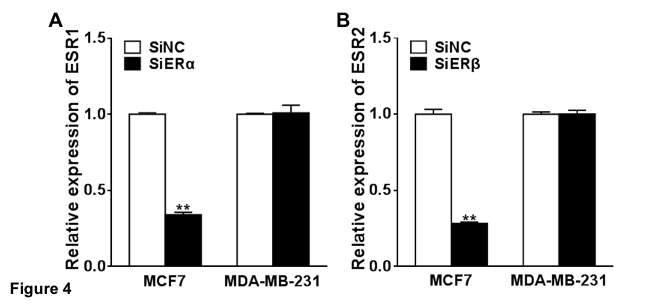

Supplement: Supplementary file 4 — Figure S4. The interference effects of siERα and siERβ in BC cells. (A, B) MCF7 and MDA-MB-231 cells were transfected with 50 nM ERα siRNAs, ERβ siRNAs or negative control siRNAs (siNC), respectively. After 48 h, the expression levels of ESR1 or ESR2 were analyzed by qRT-PCR and GAPDH levels were used as an internal control, and normalized to the value of siNC group in each cell line, respectively. ** indicates significant difference compared to the siNC group at P < 0.01. (JPEG 73 kb) [file 12943_2018_830_MOESM4_ESM.jpg]

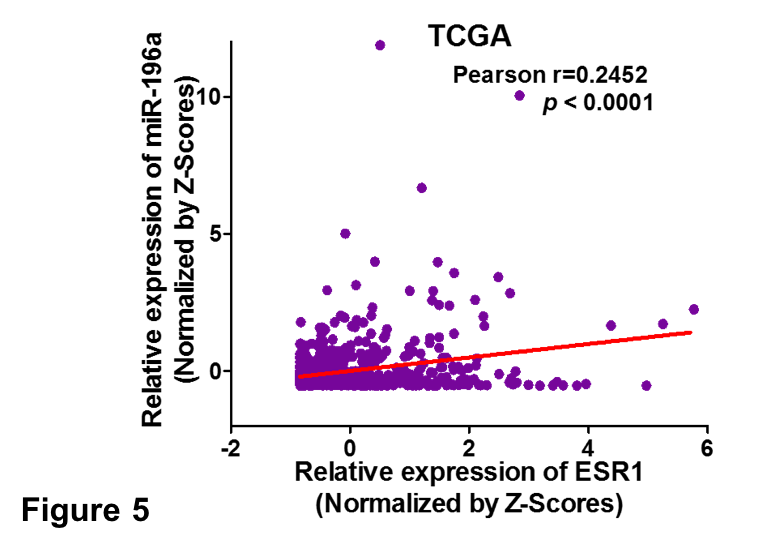

Supplement: Supplementary file 5 — Figure S5. miR-196a is positively associated with ESR1 expression levels. (A) Pearson’s correlation analysis was used to determine the correlation between the expression levels of ERα and miR-196a expression levels. (PNG 58 kb) [file 12943_2018_830_MOESM5_ESM.png]

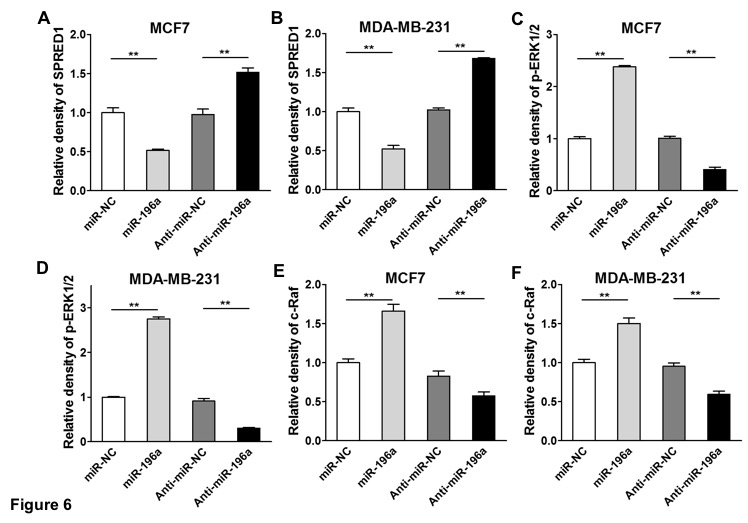

Supplement: Supplementary file 6 — Figure S6. MiR-196a directly targets and inhibits SPRED1. (A-F) Protein expression levels of SPRED1, c-Raf, pERK1/2 and GAPDH were determined using Western blot analysis in MCF7 and MDA-MB-231 cells overexpressing miR-196a, miR-NC or anti-miR-196a inhibitor and anti-miR-NC. The data were normalized as the ratio of miR-NC group, respectively. **indicated significant difference between indicated groups at P < 0.01. (JPEG 189 kb) [file 12943_2018_830_MOESM6_ESM.jpg]

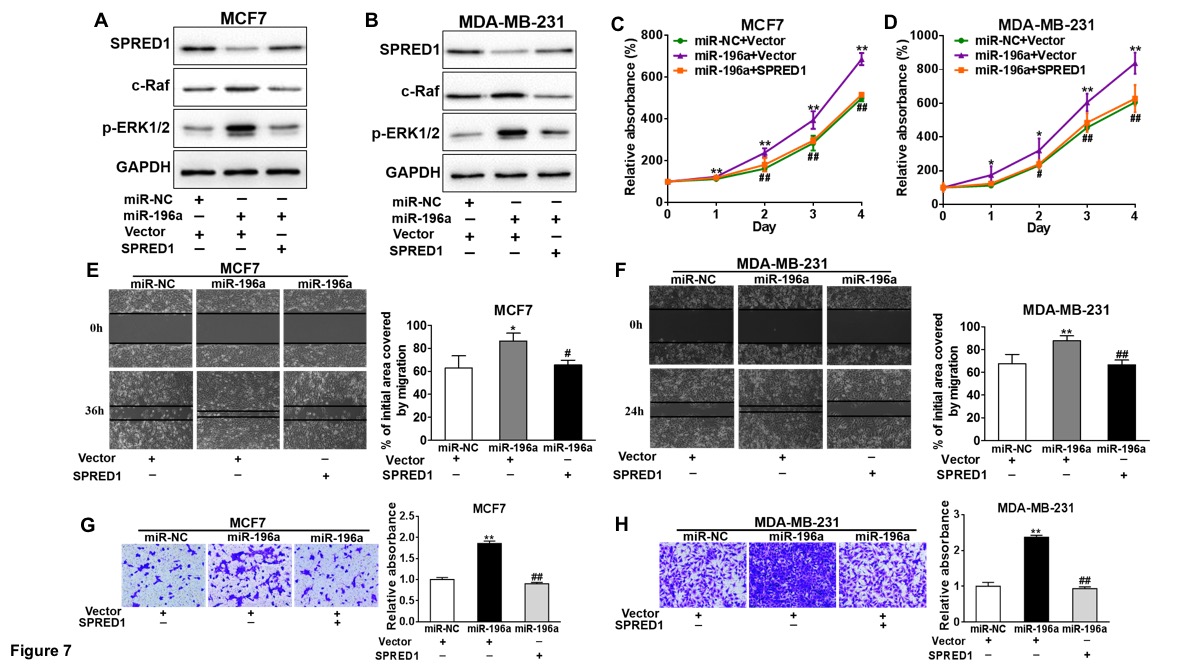

Supplement: Supplementary file 7 — Figure S7. SPRED1 reverses miR-196a-induced malignant phenotype of BC cells. MiR-196a- or miR-NC-overexpressing MCF7 cells were transfected with vector or SPRED1 cDNA without 3’-UTR. (A-B) The expression levels of SPRED1, c-Raf, pERK1/2 and GAPDH were determined by Western blot analysis after 48 h of transfection. (C-D) Cell viability was detected using CCK-8 assay. (E-F) Cells were treated and wound healing assay was performed as above. (G-H) Transwell invasion assay was performed as above using control cells and cells overexpressing miR-196a with or without SPRED1 overexpression. Data were presented as the means ± SD from three independent experiments with triple replicates per experiment. * and ** indicate significant difference between the miR-NC + Vector group and the miR-196a + Vector group with P < 0.05 and P < 0.01, respectively. # and ## indicate significant difference between the miR-196a + Vector group and the miR-196a + SPRED1 group with P < 0.05 and P < 0.01, respectively. (JPEG 552 kb) [file 12943_2018_830_MOESM7_ESM.jpg]

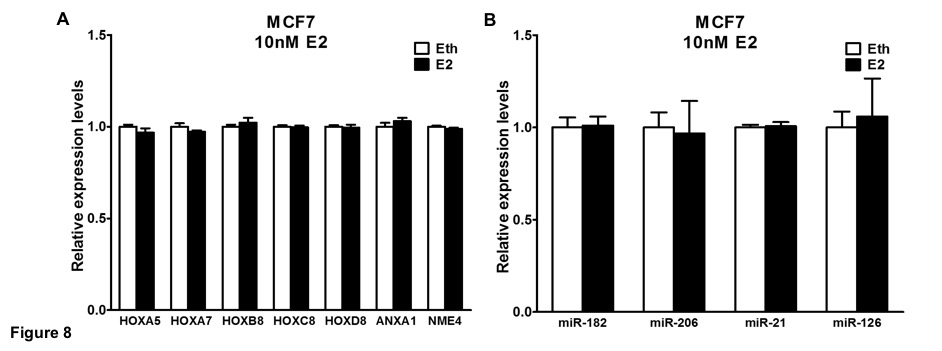

Supplement: Supplementary file 8 — Figure S8. The typical targets of miR-196a shows no change with E2 treatment in ER+ BC cells. (A) The MCF7 cells were cultured with estrogen-free medium for 72 h before E2 treatment, then treated with 10 nM E2. After 24 h, the expression levels of some reported targets of miR-196a were analyzed by qRT-PCR. (B) ER+ BC cells MCF7 were cultured with estrogen-free medium for 72 h, then treated with 10 nM E2 or equal amount of solvent Eth for 24 h. The expression levels of miRNAs which were reported to be involved in regulation of SPRED1 were analyzed by qRT-PCR and normalized to the values of the Eth control. (JPEG 105 kb) [file 12943_2018_830_MOESM8_ESM.jpg]

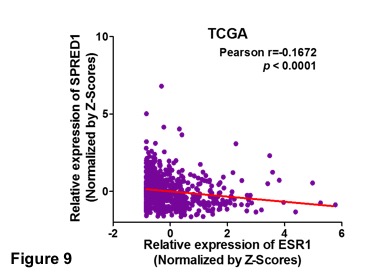

Supplement: Supplementary file 9 — Figure S9. The correlation between SPRED1 and ESR1 expression levels. (A) Pearson’s correlation analysis was used to determine the correlation between the expression levels of ERα and SPRED1 expression levels. (JPEG 62 kb) [file 12943_2018_830_MOESM9_ESM.jpg]

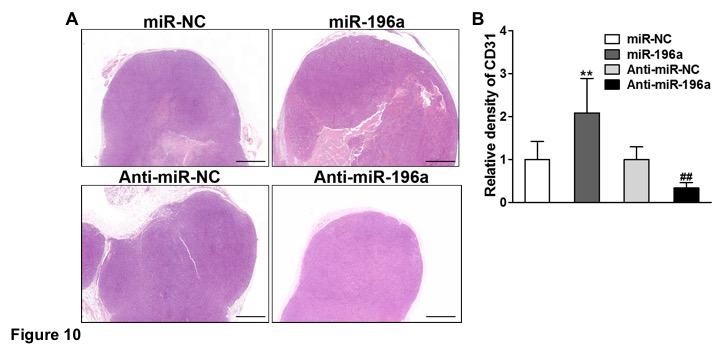

Supplement: Supplementary file 10 — Figure S10. The densities of CD31 levels were analyzed in tumor tissues with changes of miR-196a. MCF7/miR-196a, MCF7/miR-NC, MCF7/anti-miR-196a, or MCF7/anti-miR-NC cells were dispersed in 100 μl of serum-free DMEM medium and subcutaneously injected into the sides of posterior flank of nude mice (n = 4). The tumors were excised and sent to H&E (bar = 1000 μm) and immunohistochemistry to analyze the expression levels of CD31after 17 days. The densities of CD31 levels were quantified by ImageJ software, and presented as the means ± SD from 8 tumor tissues. (TIFF 1043 kb) [file 12943_2018_830_MOESM10_ESM.jpg]
